# Trace element partitioning in basaltic systems as a function of oxygen fugacity

**DOI:** 10.1007/s00410-023-02069-x

**Published:** 2023-11-27

**Authors:** J. Leuthold, J. Blundy, P. Ulmer

**Affiliations:** 1 Department of Earth Sciences, ETH Zürich, Clausiusstrasse 25, 8092 Zurich, Switzerland; 2 School of Earth Sciences, University of Bristol, Bristol, BS8 1RJ UK; 3 Department of Earth Sciences, University of Oxford, South Parks Road, Oxford, OX1 3AN UK

**Keywords:** Experimental petrology, Redox, Basalt, Clinopyroxene, Sector zoning, Trace element partitioning

## Abstract

Along with temperature, pressure and melt chemistry, magmatic oxygen fugacity (*f*O_2_) has an important influence on liquid and solid differentiation trends and melt structure. To explore the effect of redox conditions on mineral stability and mineral-melt partitioning in basaltic systems we performed equilibrium, one-atmosphere experiments on a picrite at 1200–1110 °C with *f*O_2_ ranging from NNO-4 log units to air. Clinopyroxene crystallizes from 1180 °C to near-solidus, along with plagioclase, olivine and spinel. Olivine Mg# increases with increasing *f*O_2_, eventually reacting to pigeonite. Spinel is absent under strongly reducing conditions. Mineral-melt partition coefficients (*D*) of redox-sensitive elements (Cr, Eu, V, Fe) vary systematically with *f*O_2_ and, in some cases, temperature (e.g. *D*_Cr_ in clinopyroxene). Clinopyroxene sector zoning is common; sectors along a- and b-axes have higher Al^IV^, Al^VI^, Cr and Ti and lower Mg than c-axis sectors. In terms of coupled substitutions, clinopyroxene CaTs (MgSi = Al^VI^Al^IV^) prevails under oxidized conditions (≥ NNO), where Fe^3+^ balances the charge, but is limited under reduced conditions. Overall, Al^IV^ is maximised under high temperature, oxidizing conditions and in slowly grown (a–b) sectors. High Al^IV^ facilitates incorporation of REE (REEAl^IV^ = CaSi), but *D*_*REE*_ (except *D*_Eu_) show no systematic dependence on *f*O_2_ across the experimental suite. In sector zoned clinopyroxenes enrichment in REE^3+^ in Al-rich sectors is quantitatively consistent with the greater availability of suitably-charged M2 lattice sites and the electrostatic energy penalty required to insert REE^3+^ onto unsuitably-charged M2 sites. By combining our experimental results with published data, we explore the potential for trace element oxybarometry. We show that olivine-melt *D*_V_, clinopyroxene-melt *D*_V_/*D*_Sc_ and plagioclase-melt *D*_Eu_/*D*_Sr_ all have potential as oxybarometers and we present expressions for these as a function of *f*O_2_ relative to NNO. The crystal chemical sensitivity of heterovalent cation incorporation into clinopyroxene and the melt compositional sensitivity of the Eu^2+^–Eu^3+^ redox potential limit the use of clinopyroxene-melt and plagioclase-melt, however, olivine-melt *D*_V_ affords considerable precision and accuracy as an oxybarometer that is independent of temperature, and crystal and melt composition. Variation of *D*_V_ and *D*_V_/*D*_Sc_ with *f*O_2_ for olivine and clinopyroxene contains information on redox speciation of V in coexisting melt. By comparing the redox speciation constraints from partitioning to data from Fe-free synthetic systems and XANES spectroscopy of quenched glasses, we show that homogenous equilibria involving Fe and V species modify V speciation on quench, leading to a net overall reduction in the average vanadium valence. Mineral-melt partitioning of polyvalent species can be a useful probe of redox speciation in Fe-bearing systems that is unaffected by quench effects.

**Supplementary Information:**

The online version contains supplementary material available at 10.1007/s00410-023-02069-x.

## Introduction

Magmatic oxygen fugacity (*f*O_2_) can vary significantly during magmatic differentiation and it is well known that redox exercises an important infleunce on phase relationships during basalt crystallisation (e.g. Presnall 1966; Toplis and Carroll 1995; Hammer 2006; Mollo and Vona 2014) and on melt structure, which in turn affects trace element partitioning between minerals and melts (e.g., Mysen and Virgo 1980; Kohn and Schofield 1994; Jaeger and Drake 2000; Mysen 2006; Toplis and Corgne 2002; Mysen and Dubinsky 2004; Aigner-Torres et al. 2007). Multivalent elements (e.g. S, Fe, V, Cr, Ce, Eu) are particularly sensitive to redox state, thus their partitioning behaviours have potential as oxybarometers (e.g. Luth and Canil 1993; Mallmann and O’Neill 2009, 2013; France et al. 2010).

Mineral-melt partition coefficients depend on melt chemistry and structure (e.g. Kohn and Schofield 1994; Blundy et al. 1995, 1996; Gaetani 2004; Mysen 2006), including water content (e.g. Wood and Blundy 2002; Gaetani et al 2003), crystal chemistry (e.g. Blundy and Wood 1994, 2003; Wood and Blundy 2001; Gaetani and Grove 1995; Mollo et al. 2016), temperature and pressure (e.g. Wood and Blundy 1997; Hill et al. 2011; Sun and Liang 2012). In the case of clinopyroxene, for example, major element chemistry changes with *f*O_2_, with important consequences for partitioning. In particular, the Al^IV^, Mg, and Ca contents (Ca-Tschermak’s exchange vector) exert an important influence on trace element partition coefficients (e.g. Skulski et al. 1994; Gaetani and Grove 1995; Wood and Blundy 1997; Lundstrom et al. 1998; Hill et al. 2000; Wood and Trigila 2001; Sun and Liang 2012). To avoid determination of partition coefficients for every magmatic rock at every crystallization step, thermodynamic models are used to take into account melt and crystal chemistry, pressure and temperature of crystallization and melt H_2_O content (e.g. Wood and Blundy 1997; Van Westrenen et al 2001; Hill et al. 2011; Sun and Liang 2012; Mollo et al. 2018). To date, these models do not take specific account of the effect *f*O_2_ has on both crystal chemistry, through incorporation of major structural components such as Fe^3+^, and the valence state of trace elements, such as Eu and V. We explore here the effect of redox conditions on mineral stability and trace element incorporation into clinopyroxene, olivine and plagioclase, the main mineral constituents of basaltic magmas over a wide range of *f*O_2_.

We first evaluate the effect of redox on: (i) mineral stability in a natural multi-component picrite starting material, together with a few additional experiments on a natural basalt; (ii) mineral and melt trace and major element chemistry; and (iii) mineral-melt partition coefficients. We varied both temperature and *f*O_2_ in one-atmosphere gas-mixing furnaces to explicitly quantify the effect of redox conditions on melt and crystal chemical and physical properties, with particular focus on clinopyroxene. We explored the range in redox conditions of terrestrial and extraterrestrial basaltic magmas, from four log units below nickel-nickel oxide (NNO-4) to air to test the effect of *f*O_2_ on clinopyroxene-melt REE partitioning.

## Methods

### Starting material

The picrite and basalt starting materials, identical to those employed by Leuthold et al. (2015), consist of finely ground powders of a near-primary olivine-phyric picrite dyke (B62/2; McClurg 1982; Upton et al. 2002) and a basaltic dyke (11JL33; Leuthold et al. 2014) from the Rum Layered Intrusion, Scotland. Sample B62/2 contains ~ 10 vol% euhedral to subhedral olivine phenocrysts (Fo_85.9–80.5_) in a groundmass of fine-grained olivine (Fo_78–75_), plagioclase (An_69–66_), clinopyroxene (Mg#^1^ = 0.82–0.71), Cr-spinel (Cr#^2^ = 0.32–0.43) and magnetite, with minor amphibole and mica. B62/2 is Mg-rich (12.4 wt% MgO, Mg#: 0.67) and mildly alkalic (Table 1). Sample 11JL33 (Mg#: 0.51) is a mildly alkalic (low-K) basalt (Table 1) composed of normally zoned plagioclase (An_61-15_), clinopyroxene (Mg# = 0.73–0.65), K-feldspar, magnetite with ilmenite exsolution (3 vol%), titanite, zircon, epidote, and chlorite. B62/2 picrite and 11JL33 basalt are considered as representatives of Rum parental liquids (Upton et al. 2002; Holness and Winpenny 2009; Leuthold et al. 2015) and they are very similar to those studied by Toplis and Carroll (1995) and Thy et al. (2006) in the context of the liquid line of descent of the Skaergaard layered intrusion. Recent Icelandic picrites and basalts have similar compositions to B62/2 and 11JL33 (Hémond et al. 1993). Our experimentally-determined picrite to basalt liquid line of descent (Leuthold et al. 2015; this study) thus provides information on shallow depth crystallization of the Icelandic Basalt Plateau magmas. The 11JL33 composition corresponds to that of the residual glass after ca. 40% crystallization of B62/2 (Leuthold et al. 2015). Use of 11JL33 thus represents a fractionation stage during the crystallization of B62/2 (cf. Toplis and Carroll 1995). Only our more extensive experimental dataset from the B62/2 is discussed in detail here; analyses of the 11JL33 basalt experiments are provided in the Online Resources and shown in the figures.

**Table 1:** Composition of starting materials (wt%)

	Picrite	Basalt
	B62/2^a^	11JL33^b^
SiO_2_	45.76	47.09
TiO_2_	1.56	2.14
Al_2_O_3_	13.58	14.12
Cr_2_O_3_	0.11	0.03
Fe_2_O_3_	12.38	13.21
MnO	0.19	0.2
MgO	12.42	6.47
CaO	11.25	10.43
NiO	0.04	0.01
Na_2_O	2.23	2.97
K_2_O	0.16	0.57
P_2_O_5_	0.13	0.20
LOI	0.51	2.58
Total	100.32	100.04
Mg#^c^	0.67	0.49

^a^Upton et al. (2002)

^b^Leuthold et al. (2014)

^c^Molar Mg#

### Experiments

We extend the dataset of Leuthold et al. (2015) with new experiments (#186–#255) under strongly reducing (NNO-4) and strongly oxidizing (air) conditions. We employed the same experimental technique as Leuthold et al. (2015) for all experiments reported here: the starting material powder was mixed with a small amount of ultrapure water, as a binder, and mounted on a thin Pt–Rh wire loop, melted at supra-liquidus condition, quenched and equilibrated at the desired temperature and *f*O_2_ by mixing CO_2_ and H_2_ in one-atmosphere GERO™ vertical furnaces at ETH Zürich. Experiments in air were run in the same furnace, keeping the lid open. We used the temperature oscillation technique (Mills et al. 2011; Mills and Glazner 2013; Erdmann and Koepke 2016) to grow crystals large enough for trace element analysis. The periods of oscillations were typically five rapid (10 min) cycles of cooling (10–20 °C below the target temperature) and heating (5–10 °C above the target temperature). This technique is especially efficient when oscillations are implemented just below the saturation temperature of the phase of interest. Following a period of oscillation, samples were left to equilibrate for between 5 and 91 h, depending on temperature and melt fraction, and then drop-quenched into water. Following Leuthold et al (2015) relatively short run durations were selected to minimise Fe-loss to the Pt–Rh wire. We did not observe significant differences in phase proportions or compositions between long and short runs at the same temperature-*f*O_2_. Experimental run conditions and products are provided in Online Resources 1 (B62/2) and 2 (11JL33).

### Analyses

#### Textural analysis

**Fig. 1:**
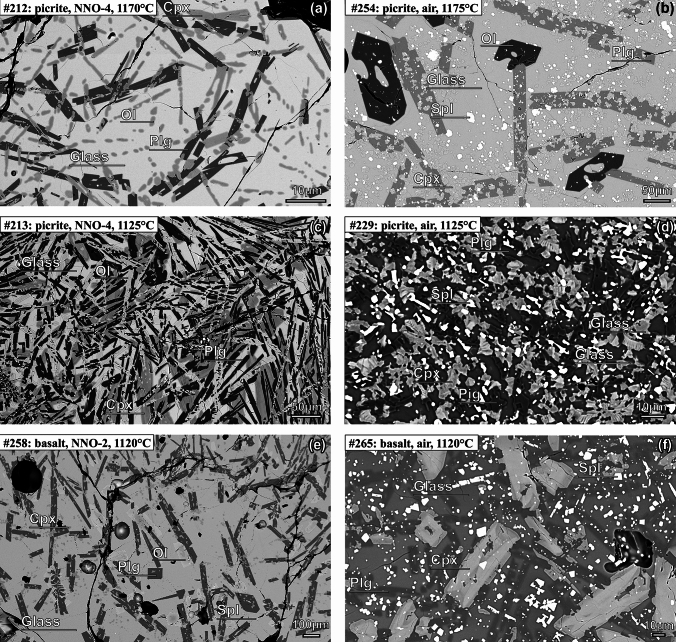
Back-scattered electron (BSE) images of selected experimental products using picritic starting material (B62/2) equilibrated close to clinopyroxene saturation temperature (**a, b**) and close to solidus temperature (**c, d**), under strongly reducing (**a, c**) and oxidizing (**b, d**) conditions. **e, f** BSE images of experimental run products using basaltic composition (11JL33). Clinopyroxene sector zoning is evident in (**f**). Glass fraction is lower under oxidizing condition (**b, d, f**). Abbreviations: Ol: olivine, Plg: plagioclase, Cpx: clinopyroxene, Spl: spinel, Pig: pigeonite. See Leuthold et al. (2015) for composition of starting materials and additional images

We used the same analytical techniques as Leuthold et al. (2015). Backscattered electron (BSE) images and X-ray maps of polished experimental runs were acquired at ETH Zürich at 20 kV with a JEOL JSM-6390 LA scanning electron microscope (SEM), equipped with a Thermo Fisher Noran energy-dispersive spectrometer (EDS X-ray detector). Selected BSE images are shown in Fig. 1 (see also Fig. 3 in Leuthold et al. 2015). We used *imageJ*™ to determine phase proportions. Repeated analyses on the same samples provide an estimate of the uncertainties on mineral proportions, which were typically less than ± 5 vol%.

#### Major elements

Analytical results are presented in Online Resource 3. We used the five-spectrometer ETH Zürich JEOL JXA-8200 Electron Probe Microanalyser (EPMA) for major element analyses at 15 kV and 20 nA and a beam size of 1 μm for crystals and 1–10 μm for glass. Natural and synthetic silicates and oxides were used as standards: wollastonite (Ca, Si); aegirine (Na), microcline (K), fayalite (Fe); forsterite Mg); corundum (Al); apatite (P); chromite (Cr), pyrolusite (Mn), rutile (Ti) and bunsenite (Ni). Peak (background) times were 20 s (10 s) for all elements except Na and K (10/5 s) and Ni and Cr (30/15 s). Internal standards were regularly analysed as unknowns (typically every 60–100 analyses) and checked for drift < 1.5%. We found no variation in major element concentrations from varying the spot size on glass. Great care was taken to avoid contamination by fluorescence from the surrounding glass or inclusions and poor analyses were discarded. Analytical uncertainty is typically less than 1.0% relative. This is important when calculating the pyroxene structural formulae (calculated on a four cations basis), as even small errors on Si or Na control the charge deficit and hence the stoichiometrically calculated amount of Fe^2+^ and Fe^3+^ (e.g. Sobolev et al. 1999; Borisov et al. 2017). To further limit analytical errors, one spectrometer was dedicated to the analysis of Si alone, to limit drift during the analytical sequence due to movement of the TAP crystal. Na was measured for 10 s at the beginning of the sequence, to limit elemental migration. Reasonable analytical errors on SiO_2_ and Na_2_O (i.e. 0.5 wt% and 0.15 wt% respectively) are lower than errors due to experimental reproducibility.

In terms of analytical precision a 1% relative variation on SiO_2_ and FeO analyses results in a change of the calculated clinopyroxene Fe^3+^/Fe_tot_ ratio by ± 0.06 and ±  < 0.01 respectively. The effect on the stoichiometry of VO_2_ or V_2_O_3_ (< 0.005 apfu) and P_2_O_5_ (< 0.001 apfu) are negligible (change in Fe^3+^/Fe_tot_ ≤ 0.01). It has been suggested that, in Si-depleted clinopyroxene, some Fe^3+^ might enter the clinopyroxene tetrahedral site (Virgo 1972; Rossman 1980; Akasaka 1983), however we observe no clear correlation between SiO_2_ and Fe^3+^ and consider only Si and Al to occur on the tetrahedral site of our experimental augites.

EPMA analyses reveal Fe- and Ni progressive loss to the Pt–Rh wire during the experiment, especially under reducing conditions. At *f*O_2_ ≤ NNO-3), the 70 µm Pt–Rh wire becomes Fe-saturated within a few hours. Fe and Ni losses are limited to < 0.2 wt% FeO and < 0.01 wt% NiO by a high sample/loop volume ratio. Mass balance calculations reveal bulk FeO_tot_ decreases from 11.4 wt% in short supra-liquidus experiments to ca. 11.4–9.3 wt% in most long, low-temperature runs, with no distinct effect of *f*O_2_. Since the study of Leuthold et al. (2015) we have discovered that sector zoning (Fig. 1f) exerts an important control on clinopyroxene Al_2_O_3_ and Cr_2_O_3_ concentrations. In this study both sectors were analyzed separately. Neave et al. (2019) suggested only bright sectors represent thermodynamic equilibrium compositions.

A few experiments in Leuthold et al. (2015) were cooling-rate (5–30 °C/h) experiments. Rim analyses in equilibrium with the surrounding glass were considered. The apparent partitioning of major elements (CaO, MgO, FeO) between pyroxene and basaltic melt is independent of cooling rate and depends only upon the quenching temperature (Gamble and Taylor 1980). However, Hammer (2006) pointed to similar core compositions but stronger zoning under oxidizing conditions and at slow cooling. Mollo et al. (2010, 2011) observed an increase in anorthite content in plagioclase and in clinopyroxene Fe^3+^/Fe_tot_ and Al^IV^ at higher cooling rate (30–900 °C/h) that in turn were found to affect trace element partitioning (Mollo et al. 2013). Our cooling-rate experiments were duplicated with equilibrium experiments and no systematic difference in modal abundance or chemistry of glass and minerals (clinopyroxene, plagioclase) was observed. However, olivine rims show strong normal zoning in cooling rate experiments.

#### Trace elements

Glass and crystals were analyzed by LA-ICP-MS at ETH Zürich using a Thermo Element XR mass spectrometer connected to a 193 nm Resonetics ArF Excimer laser. The laser was operated in a Laurin Technic S155 ablation cell with a spot size between 13 and 20 µm (rarely 30 µm for some glass analyses), frequency of 2–5 Hz and laser power density of 2 J cm^−2^. Individual analyses were 5–30 s duration. Each analysis spot was carefully checked for absence of inclusions. Extra care allowed analyses of separate clinopyroxene sectors in many cases. EPMA data were used as internal standards for all measured minerals (Ca for pyroxene and plagioclase, Mg for olivine) and glasses (Ca). NIST SRM610 was used for external standardization and GSD-1G basalt glass as secondary standard. Raw data were reduced off-line using the SILLS software (Guillong et al. 2008). 1σ uncertainty for V is typically 0.1 rel% and error on secondary standard GSD-1G is < 5–10 rel% (< 5 rel% for ≥ 20 µm spots). 1σ uncertainties for REE range between 0.4 and 1.5 rel% and reproducibility of GSD-1G is < 6 rel%. For major oxides analysed by EPMA and LA-ICP-MS and not used for internal calibration agreement between the two techniques has an absolute average relative deviation of 12% for TiO_2_, 13% for Al_2_O_3_, 10% for FeO and 9% for MnO across a very wide range of concentrations. For glass only the absolute average relative deviation is 6.5% for TiO_2_, 4.7% for Al_2_O_3_, 6.7% for FeO and 6.3% for MnO, which is within the expected uncertainties based on the secondary standard basalt glass. The largest deviations between EPMA and LA-ICP-MS were observed for Al_2_O_3_ and TiO_2_ in a few sector-zoned clinopyroxenes where the larger analytical volume for LA-ICP-MS, the small individual sector dimensions and the possible presence of very fine scale concentric zoning compromises agreement between the two techniques. For these experiments (run129/1, run171, run192, run272) trace element partitioning data are interpreted with caution.

We calculated clinopyroxene-, olivine and plagioclase-melt trace element (*i*) weight fraction Nernst partition coefficients ($D_i$). Clinopyroxene *D*_Cr_ was calculated using EPMA analyses, except for glass analyses below the limit of detection, typically found at low temperature and oxidizing conditions, which were calculated using LA-ICP-MS analyses (EPMA and LA-ICP-MS Cr analyses show a 1:1 correlation with a R^2^ of 0.90). Full analytical results are presented in Online Resource 3.

## Phase proportions and compositions

### Glass

Glass proportion remains high from the liquidus (ca. 1300 °C) until plagioclase saturation at 1190 °C (at NNO-0.8; > 83 vol% glass; Online Resource 4). Subsequently, glass proportion decreases regularly by ca. 10 vol% per 10 °C. At low melt fraction, glass proportion at a given temperature is slightly lower under oxidizing conditions than reducing conditions (Online Resources 4 and 5). Thus, the effective solidus (i.e. melt fractions lower than about 5%, which is the minimum that can be assessed experimentally) temperature is estimated to be 1115 °C in air and 1090 °C under reducing conditions. In 11JL33 basalt experiments at NNO-0.8, olivine, spinel and plagioclase co-saturate at 1165 °C, followed by clinopyroxene at ca. 1150 °C; the effective solidus temperature is 1050 °C.

**Fig. 2:**
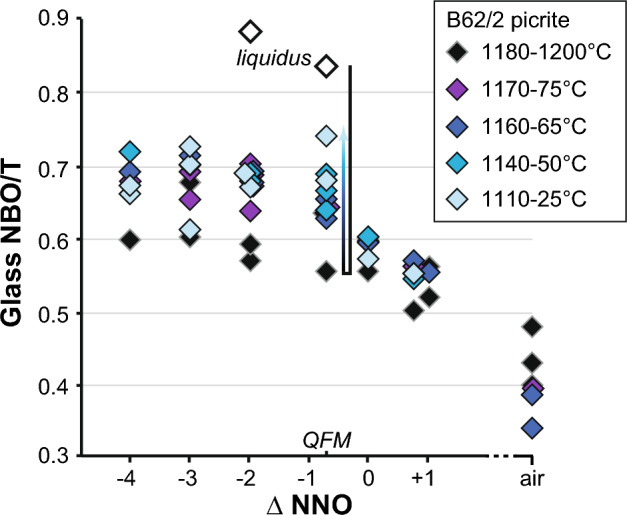
NBO/T of experimental glasses as a function of *f*O_2_ (ΔNNO). The melt polymerizes upon cooling from the liquidus temperature to plagioclase saturation and becomes depolymerized thereafter. The melt is distinctly more polymerized under oxidizing conditions, where Fe^3+^/Fe_tot_ is high. Open symbols denote experimental starting material glasses: B62/2 (picrite) and 11JL33 (basalt)

Oxygen fugacity changes melt chemistry and hence melt structure. Glass NBO/T decreases from 0.83 to 0.55 from the liquidus to plagioclase saturation and increases slightly thereafter (0.74 at 1110 °C, NNO-0.8; Fig. 2), due to the increase in network modifying alkalis (Na and K) inducing depolymerization [i.e. increasing NBO/T] (Borisov et al. 2017) upon cooling. At constant temperature (1175–1160 °C), where olivine + plagioclase + clinopyroxene are co-saturated, glass NBO/T is almost constant from strongly reducing conditions (ca. 0.68 at NNO-4) to NNO-0.8 (ca. 0.66) and then decreases sharply with further increase in *f*O_2_ (ca. 0.34–0.48 in air), due to the crystallization of abundant ülvospinel and increased Fe^3+^/Fe_tot_ ratio (Fe^3+^ acts as a network-former, lowering NBO/T; Mysen 2006) (Fig. 2). NBO/T decrease at ≥ NNO is sharpened by the additional effect of lower liquid fraction under oxidizing conditions (Online Resource 4).

#### Major element chemistry

Picritic starting material differentiates to basaltic glass during equilibrium crystallization. Glass Fe^3+^ was estimated using the Kress and Carmichael (1991) algorithm at the experimental temperature and redox conditions. Upon cooling along oxygen buffers, glass Fe^3+^/Fe_tot_ is maximal at ca. 1180 °C, increasing from ca. 0.02 (i.e. Fe^3+^/Fe^2+^ of 0.04) at NNO-4 to 0.17 at NNO + 1 and 0.35 in air (i.e. Fe^3+^/Fe^2+^  = 1.04). Olivine, plagioclase and clinopyroxene crystallization drives melt compositions towards high Fe-content along a tholeiitic differentiation trend (e.g. Grove and Baker 1984 and references therein; Hammer 2006). There is a turnover when magnetite-ülvospinel stability is reached and its modal proportion (up to ca. 4–12 vol% in air, at 1200–1125 °C, Online Resources 1 and 2) increases under oxidising condition (Online Resources 4 and 5). As a consequence, melt SiO_2_ and MgO are enriched while Fe enrichment is inhibited (e.g. Hammer 2006; Toplis et al. 1994; Toplis and Carroll 1995), resulting in higher Mg# and differentiation along a quartz-normative calc-alkaline differentiation trend (Grove and Baker 1984 and references therein; Hammer 2006). This effect is partly counterbalanced by increased clinopyroxene and pigeonite abundance relative to olivine.

**Fig. 3:**
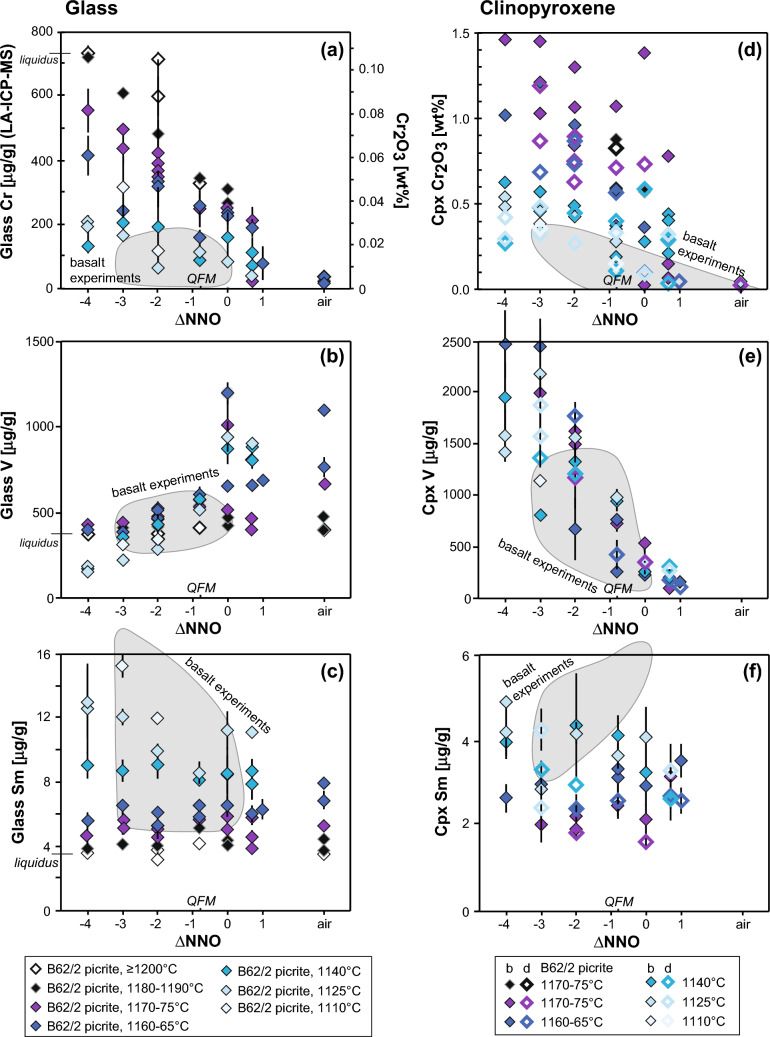
Trace element variations in experimental run products for three representative trace elements. Cr_2_O_3_ (**a, d**), V (**b, e**) and Sm (**c, f**) variation with *f*O_2_ in glass (**a–c**) and clinopyroxene (**d–f**). Cr_2_O_3_ shows strong variations due to *f*O_2_, but also to temperature and sector zoning. Clinopyroxene V concentration shows little variation due to temperature and no variation due to mineral structure, but strong variation due to *f*O_2_. Melt and clinopyroxene Sm concentrations increase upon cooling and differentiation but show no distinct variation due to *f*O_2_. Error bars are 1 s.d. Individual picrite (B62/2) experimental data plotted; shaded fields show data ranges for basalt (11JL33) experiments. In d, e, f, filled symbols are bright sectors (slowly grown faces along the a- and b-axes) and open symbols are dark sectors

Glass Cr_2_O_3_ varies from 0.11 wt% on the liquidus down to 0.01 wt%, after Cr-spinel saturation and subsequent clinopyroxene crystallization (Leuthold et al. 2015). At constant temperature (1175–1160 °C), glass Cr is constant at ca. 470 μg/g (0.07 wt% Cr_2_O_3_) from NNO-4 to NNO-2, where little or no Cr-spinel crystallizes, but decreases down to 20 μg/g in air (Fig. 3a). Under oxidizing conditions, clinopyroxene and ülvospinel *D*_Cr_ are lower, resulting in limited glass Cr_2_O_3_ variation upon cooling. V and Cr have similar behavior. Under reducing condition (NNO-4), glass V concentration decreases down temperature from 400 at 1190 °C to 150 μg/g at 1125 °C (Fig. 3b). The opposite trend is observed under oxidizing conditions; glass V increases from 400 at 1200 °C to ~ 1100 μg/g at 1165–1125 °C, due to lower clinopyroxene and spinel *D*_V_. The V content in glass is identical in experiments using the basaltic starting material. The glass V/Sc ratio, used to estimate basalt *f*O_2_ and discriminate between geodynamical settings (e.g. Bucholz and Kelemen 2019), increases under oxidizing condition (i.e. V/Sc increases from 10 to 4 during cooling at NNO-4 and 10–30 at ≥ NNO). Glass TiO_2_ concentration increases steadily from ~ 1.8 wt% at 1200 °C to ~ 6.8 wt% at 1110 °C below NNO and to ~ 4.2 wt% at ≤ 1140 °C above NNO, when ülvospinel saturates. Glass Al_2_O_3_, MgO, CaO, Na_2_O and K_2_O are unaffected by redox conditions, at constant liquid fraction (Online Resource 3).

#### Trace element chemistry

Glass REE content, as exemplified by Sm, increases by a factor of ~ 4 upon cooling to 1110 °C (Fig. 3c), consistent with incompatible behaviour up to 80% equilibrium crystallization (Leuthold et al. 2014). There is no discernible effect of *f*O_2_ on glass REE concentration (Figs. 3c). We confirm observations by Wilke and Behrens (1999) and Aigner-Torres et al. (2007) that the glass Eu concentration and Eu/Eu* are distinctly lower under reducing conditions, due to higher plagioclase/glass *D*_Eu_. Sc decreases regularly, Sr slightly increases and Zr increases strongly upon cooling, with no effect of *f*O_2_. Glass Ni content is slightly higher above NNO (ca. 112 μg/g) than under reduced conditions (ca. 88 μg/g), as a result of diminished olivine stability.

### Olivine

Olivine is a liquidus phase, together with spinel, with a saturation temperature close to 1300 °C at NNO-0.8 (Online Resources 4 and 5). Olivine grains in our experiments frequently show skeletal form (Fig. 1), due to fast growth from olivine super-saturated melt. However, the *D*_Fe2+-Mg_ (with melt Fe^3+^/Fe_tot_ calculated as described above) is constant at 0.292 ± 0.029 (independent of temperature and *f*O_2_), within error of the one atmosphere canonical values of 0.300 ± 0.002 obtained by Ulmer (1989) for experiments on a picrobasalt (higher MgO than B62/2) and 0.312 ± 0.001 proposed by Blundy et al (2020) on the basis of a large multi-composition experimental dataset with measured glass Fe^3+^/Fe_tot_. We thus infer that chemical equilibrium was closely approached. Olivine modal abundance is ca. 10 vol% of the magma when plagioclase saturates at 1200 °C and reaches ca. 17 vol% (< 22 vol%) below 1170 °C. It is lower under strongly oxidised conditions (≥ NNO + 0.7) (as also documented by Roeder and Emslie 1970; Hammer 2006), due to low melt NBO/T at high Fe^3+^/Fe_tot_ and increased SiO_2_ activity as a result of abundant spinel crystallization. In air, olivine is absent close to the solidus (Fig. 1d and Online Resource 5).

#### Major element chemistry

Olivine FeO content increases and forsterite (Fo) content decreases upon cooling at fixed *f*O_2_ (relative to NNO), e.g. from Fo_83_ at 1190 °C to Fo_70_ at 1120 °C at NNO-3. As the olivine structure accommodates very little trivalent cations (Fe^3+^, Cr^3+^, V^3+^), the melt’s low Fe^2+^ content under oxidized condition is responsible for a strong isothermal increase of olivine forsterite content (cf. Roeder and Emslie 1970; Mysen 2006; Toplis and Carroll 1995; Davis and Cottrell 2018) that is greater than its total range from liquidus to solidus along a given buffer. For example, at 1190 °C forsterite increases gradually from Fo_84_ at NNO-4 to Fo_87_ at NNO + 1 and then abruptly to Fo_98_ in air. For *f*O_2_ below NNO + 1 the gradient in Fo with *f*O_2_ is similar to that observed at 1225 °C by Davis and Cottrell (2018) in a basaltic starting composition.

#### Trace element chemistry

Olivine CaO and *D*_Ca_ are constant upon cooling in equilibrium experiments and gradually decrease under oxidizing conditions (0.6 wt% at NNO-4 to 0.22 wt% in air). Olivine Al_2_O_3_ content, although proposed as a thermometer by Coogan et al. (2014), is invariant with temperature and *f*O_2_. At QFM condition, Karner et al. (2008) determined that 70% of the redox-sensitive V in olivine occurs as V^3+^ (the remaining as V^4+^). V decreases from ca. 100 μg/g at NNO-4 to ca. 30–15 μg/g at ≥ NNO-0.8 and < 5 μg/g in air, as observed in previous studies for a range of mafic magma systems, e.g. komatiite (Canil 1997; Mallmann and O’Neill 2013), picrite (Canil and Fedortchouk 2001), CMAS (Mallmann and O’Neill 2009; 2013) and MORB (Mallmann and O’Neill 2013). There is no effect of pressure, temperature or compositions on olivine/glass *D*_V_ (Canil and Fedortchouk 2001), but *D*_V_, as observed by Mallmann and O’Neill (2009, 2013) decreases with increasing *f*O_2_ due to the increasing proportion of less compatible V^4+^ (and eventually V^5+^) in the melt (0.5 at NNO-4, 0.03 at *f*O_2_ ≥ NNO-0.8 and ca. 0.01 in air). Olivine Cr_2_O_3_ contents decrease strongly both at high *f*O_2_ and at low temperature (700 to 20 μg/g at 1160–75 °C from NNO-4 to air respectively; ca. 300 to < 70 μg/g at 1125–40 °C) due to the competing effects of spinel, but *D*_Cr_ remains constant at ca. 1.1. Olivine Ni concentration increases from NNO-4 (ca. 1200 μg/g, at 1160–1175 °C) to NNO + 1 (ca. 2400 μg/g, at 1160–1175 °C) and decreases upon cooling (by a factor of 1.5–2 from 1200 to 1125 °C). *D*_Ni_ (from 12 up to 28; similar to Li and Ripley 2010) show little dependence on *f*O_2_ or temperature. *D*_Sc_ ranges from 0.15 to 0.47. It decreases with increasing Fo content from Fo_60_ to Fo_98_ due to the mismatch between the ionic radius (in VI-fold co-ordination) of Sc^3+^ (0.745 Å) with Mg^2+^ (0.720 Å) and Fe^2+^ (0.780 Å; Shannon 1976); at intermediate Fo contents Sc^3+^ is very close in ionic radius to the weighted average of Mg^2+^ and Fe^2+^. However, *D*_Sc_ also increases with increasing temperature and decrease with increasing *f*O_2_ relative to NNO. These apparent effects are simply a consequence of the aliasing of Fo with temperature and *f*O_2_ in our experimental dataset.

### Spinel

Spinel was studied in detail by Leuthold et al. (2015) and their main findings are summarized here. Upon cooling, spinel chemistry varies from Cr-spinel to magnetite-ülvospinel s.s., with strong enrichment in Fe^3+^ and TiO_2_ and decrease in Al, Cr and Mg. Under oxidizing conditions, where coexisting liquids are characterized by high Fe^3+^ contents, ülvospinel-magnetite stability is strongly increased, while spinel is absent under strongly reducing conditions (≤ NNO-3) (Fig. 1 and Online Resource 5). Spinel V concentration increases slightly upon cooling and from oxidizing to reducing conditions (0.9 wt% at NNO-3 to 0.1 wt% at NNO + 1, at 1180 °C). We have only a few data on spinel/glass *D*_V_, which consistently show a decrease under oxidizing condition (ca. 13 at NNO-2 to < 2 at NNO + 1) and point to a minor effect of temperature. Canil (2002) showed temperature, pressure, or melt composition have no effect on spinel/glass *D*_V_. However, spinel *D*_V_ strongly depends on the bulk system Cr/Al ratio, which is constant in our equilibrium crystallization experiments but can be rather variable for natural mafic magmas. Spinel NiO concentration is nearly constant at 0.12–0.31 wt% at ≤ NNO + 1, increasing to ca. 0.38 in air.

### Plagioclase

Plagioclase saturates at 1190 and 1195 °C under reducing and oxidizing conditions respectively (Online Resources 4 and 5). As a polymerized phase, plagioclase is stabilized under oxidizing conditions, where the melt is more polymerized and has a higher crystallinity. The modal abundance strongly increases upon cooling (up to ca. 50 vol% of the crystal assemblage).

#### Major element chemistry

Under reducing condition, *f*O_2_ has no influence on the plagioclase anorthite (An) content (An_~76_ at NNO-4 to NNO-0.8 at 1175–1160 °C; An_69_ at 1110 °C). Plagioclase An is a function of *f*O_2_ under oxidizing conditions (An_75_ at NNO to An_66_ in air) at constant temperature (1175–1160 °C) and decreases down to An_58_ at 1125 °C in air. The lower plagioclase An content at high *f*O_2_ is most likely a result of lower melt fraction and enhanced clinopyroxene stability, depleting the melt in CaO relative to Na_2_O.

**Fig. 4:**
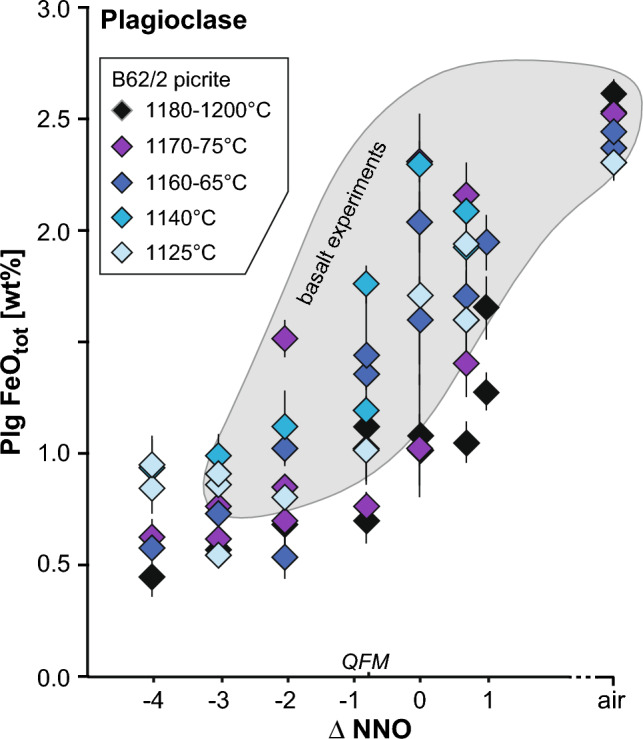
Plagioclase FeO_tot_ content as a function of *f*O_2_ (ΔNNO) showing a strong increase at high *f*O_2_ due to preferential incorporation of Fe^3+^. Based on our experiments, temperature and crystallization have lesser effect. Individual picrite (B62/2) experimental data plotted; shaded fields show data ranges for basalt (11JL33) experiments

Plagioclase FeO_tot_ increases from 0.4 to 1.0 wt% (1190–1125 °C) at ≤ NNO-3 to 2.6–2.3 wt% (1195–1125 °C) in air (Fig. 4), as previously documented by Phinney (1992), Toplis and Carroll (1995), Lundgaard and Tegner (2004) and France et al. (2010). In the Nandedkar et al. (2014) 0.7 GPa fractional crystallization experiments from a near-primary olivine-tholeiite, plagioclase FeO increases until saturation of ülvospinel and magnetite, whereupon it decreases abruptly. In the Mollo et al. (2018) titanomagnetite-saturated experiments on Etna starting compositions, plagioclase FeO concentration decreases from basaltic to trachybasaltic to basaltic trachyandesitic starting compositions. Upon cooling, plagioclase FeO_tot_ concentration increases under reducing conditions, where spinel abundance is low, and decreases under oxidizing conditions, where abundant ülvospinel co-crystallizes. The effect of *f*O_2_ on plagioclase FeO_tot_ is significantly greater than that of temperature (Fig. 4).

#### Trace element chemistry

Plagioclase TiO_2_ is used in gabbroic rocks to estimate parental magma chemistry (Thy et al. 2006; Humphreys 2011; Leuthold et al. 2018), so testing the specific role of *f*O_2_ is important in that respect. Plagioclase TiO_2_ content gradually increases upon cooling (0.08–0.27 wt%), independent of *f*O_2_. Phinney (1992) found no significant change in *D*_Ti﻿_ over an *f*O_2_ variation of 13 orders of magnitude. Our measured *D*_Ti﻿_ only decreases slightly (± 1 s.d.) upon cooling (from ca. 0.04 at 1175 °C to ca. 0.03 at 1125 °C) alongside the decreasing anorthite content (*D*_*Ti*_ = 0.04 ± 0.01 from picrite B62/2 to basalt JL33 starting material). Titanium in plagioclase is thus an appropriate element to calculate parental melt TiO_2_ content in basaltic systems even under unknown or variable *f*O_2_ conditions.

Sr, Ba and LREE (La, Ce, Eu) were the only plagioclase trace elements measured with confidence by LA-ICP-MS. Sr, Ba and LREE show a distinct increase with decreasing anorthite content upon cooling, consistent with Dohmen and Blundy (2014), but *D*_LREE﻿_ decreases towards lower temperature. *f*O_2_ has no distinct effect on *D*_Sr﻿_, *D*_Ba﻿_ or *D*_LREE﻿_. Plagioclase Eu content increases upon cooling, especially under reducing conditions. Wilke and Behrens (1999) and Aigner-Torres et al. (2007) showed a strong relation between *f*O_2_ and *D*_Eu_, due to the higher compatibility of Eu^2+^ (similar ionic radius to Sr) over Eu^3+^ in the plagioclase structure. *D*_Eu_ remains constant upon cooling but varies regularly from ca. 0.5 at NNO-4 to 0.05 at NNO + 1. Eu in plagioclase is below the detection limit under more oxidised conditions, but we can make an estimate of *D*_Eu_ using the lattice strain model of Dohmen and Blundy (2014) with the measured values of *D*_La_, *D*_Ce_, *D*_Pr_ and *D*_Y_ and assuming that all Eu is trivalent at these conditions. The calculated *D*_Eu_ for two B62/2 experiments (254 and 48) run in air is 0.015.

### Clinopyroxene

**Fig. 5:**
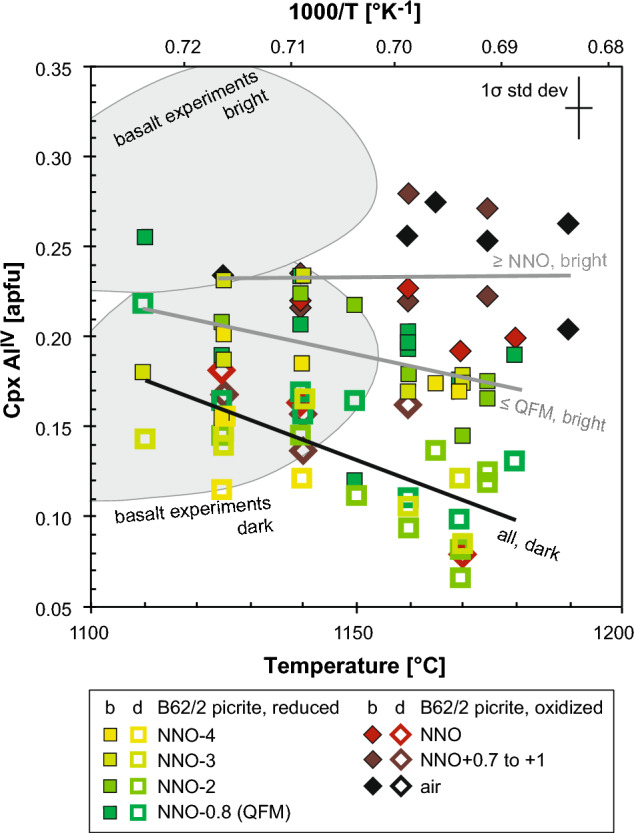
Clinopyroxene tetrahedral aluminium (Al^IV^ atoms per formula unit) as a function of run temperature (°C) decreases upon cooling under relatively reducing (*f*O_2_ < NNO) conditions. Al^IV^ is significantly higher in bright sectors of sector zoned clinopyroxene (slowly grown faces along the a- and b-axes). Individual picrite (B62/2) experimental data plotted; shaded fields show data ranges for basalt (11JL33) experiments. Filled symbols are bright sectors; open symbols are dark sectors

**Fig. 6:**
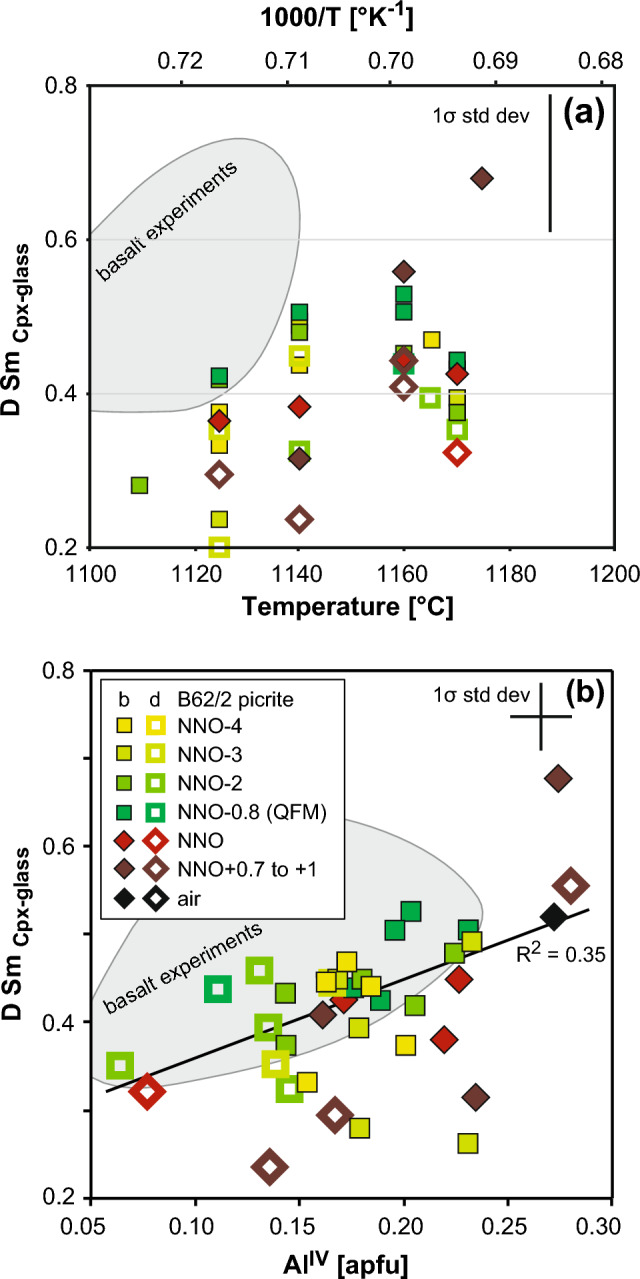
Samarium partitioning in clinopyroxene. **a*** D*_Sm﻿_ clinopyroxene as a function of temperature (°C) and reciprocal temperature (K^−1^).* D*_Sm﻿_ decreases upon cooling in equilibrium experiments and increases with differentiation from picrite to basalt (shaded field). **b** Clinopyroxene-glass* D*_Sm﻿_ as a function of Al^IV^ (assuming only Si and Al on tetrahedral site) reveals positive correlation with calculated clinopyroxene Al^IV^ (which is itself strongly correlated to the Fe^3+^ in the structure)

Clinopyroxene saturates from ca. 1170 °C at NNO-4 to ca. 1195 °C in air (Online Resource 5) due to stabilization by higher Fe^3+^ in the melt (Oba and Onuma 1978; Onuma 1983), when olivine modal abundance stops increasing. Clinopyroxene represents ca. 20 vol% of the crystal assemblage at 1160 °C and ca. 30 vol% at 1125 °C. It is less abundant under reducing conditions (Online Resource 4), confirming observations by Toplis and Corgne (2002) and Oba and Onuma (1978). Crystals frequently show sector zoning (Fig. 1f). In such situations extra care was necessary when reducing EPMA and LA-ICP-MS analyses and individual sectors were analysed whenever possible. Skulski et al. (1994) and Schwandt and McKay (2006) showed fractionation of trace elements between different sectors. We observed higher Al (and Al^IV^/Al_tot_), Ti, Cr, Fe (with no effect on Fe^3+^/Fe_tot_), Ca, Ni and LREE concentrations in higher-Z (bright BSE) slowly grown sectors (a- and b-axes), and higher Si, Mg, Sr and Zr in lower-Z (dark BSE) sectors grown along the clinopyroxene long c-axis (Figs. 3a, 5 and 6), similar to Skulski et al. (1994).

#### Chemistry

The effects of *f*O_2_ on clinopyroxene major element concentrations are well known (e.g. Lundstrom et al. 1998; Toplis and Corgne 2002), i.e., under oxidizing conditions, Al^IV^, Fe^3+^, Mn and Na increase, while Si, Al^VI^, Cr, V, Fe^2+^ and Ca decrease in both sectors. However, studies focusing on the effect of *f*O_2_ on clinopyroxene/basalt Fe, Cr and V partition coefficients under conditions prevailing on Earth are rare (e.g. Mallmann and O’Neill 2009; Bédard 2014; Shepherd et al. 2022) and the present study is the first to explore systematically the effect of *f*O_2_ on *D*_REE﻿_.

#### Aluminium

At saturation temperature, clinopyroxene Al^IV^ is lowest in dark sectors under reducing and oxidizing condition (ca. 0.11 apfu), low under reducing condition in bright sectors (ca. 0.18 apfu at ≤ NNO-0.8) and high under oxidizing condition in bright sectors (ca. 0.24 apfu at ≥ NNO + 0.8) (Fig. 5). The uncertainty on Al^IV^ is, however, large (s.d. is ± 0.02 apfu; error due to 1 wt% error on SiO_2_ measurement is 0.02). Nevertheless, analytical resolution is sufficient to resolve the difference between strongly reducing and strongly oxidizing conditions. Additionally, duplicate and triplicate experiments are coherent and confirm the variations described here. We thus infer that Al^IV^ increases from reducing to oxidizing conditions and in slowly grown bright sectors. Similar observations are true for 11JL33 basalt starting material. Mollo et al. (2010) observed distinctly higher Al^IV^ in rapidly cooled clinopyroxene (> 30–900 °C/h). We see no such differences between crystals grown in isothermal experiments and clinopyroxene rims crystallized in cooling rate experiments (5–30 °C/h). The growth rate in cooling experiments was therefore low enough to avoid appreciable disequilibrium.

In our NNO-4 to air experiments at ca. 1170 °C, Al^IV^ in bright sectors increases from 0.07 to 0.27 apfu (Fig. 5), while Fe^3+^ (calculated by stoichiometry) increases from < 0.08 apfu (≤ NNO-2) to 0.08–0.21 apfu (≥ NNO). At *f*O_2_ ≥ NNO, Al^IV^ is positively correlated with Fe^3+^ (*R* = 0.79) on the M-site, close to a 1:1 ratio, inferring Ferri-Aluminium Tschermak’s (FATs) substitution, with increasing fassaitic component. Mollo and Vona (2014) observed that the Si/Al ratio depends strongly on the *f*O_2_ of the system and higher Fe^3+^ contents in clinopyroxene facilitate the substitution of Al^IV^ for Si in the tetrahedral site. Under reducing condition. 2Al^IV^ + Ti substitute for 2Si + Mg. There is little Al^IV^ increase with pressure in natural mafic systems (Hill et al. 2011; Bédard 2014; Hirschmann et al. 2008 [LEPR]).

##### Iron and Magnesium

FeO_t_ increases from reducing to oxidizing conditions and upon cooling under reducing conditions (≤ NNO). It is invariant with temperature under more oxidizing conditions. There is a strong and regular Fe^3+^ (calculated from stoichiometry) increase at high *f*O_2_, with Fe^3+^/Fe_tot_ increasing from ca. 0.14 at NNO-4 to ca. 0.94 in air; Na also increases from 0.24 to 0.40 wt% along the acmite vector. For comparison, Luth and Canil (1993) presented an oxybarometer based on the reaction 4 CaFe^3+^AlSiO_6_ (FATs) + 3 Fe_2_Si_2_O_6_ (ferrosilite) = 2 CaAl_2_SiO_6_ (CaTs) + 2 CaFeSi_2_O_6_ (hedenbergite) + 4 Fe_2_SiO4 (fayalite) + O_2_, where the clinopyroxene Fe^3+^/Fe_tot_ ratio varies from 0.15 to 0.38 at NNO-0.8 to 0.03–0.07 at NNO-4. Our results are consistent with those predictions. Clinopyroxene Fe^3+^/Fe_tot_ remains constant within error upon cooling, which we ascribe to buffering by crystallization of Fe^3+^-rich spinel. Fe^3+^/Fe_tot_ (and hence FATS) is not fractionated between sectors.

At *f*O_2_ ≤ NNO, clinopyroxene MgO gradually decreases upon cooling, but remains invariant above NNO. MgO is constant with *f*O_2_ at 1160–1170 °C, but increases under oxidizing conditions (≥ NNO) at 1125–1140 °C. Mg apfu is anticorrelated with Al^IV^, Al^VI^, Fe^2+^ and Ti. As a consequence, the apparent Mg# (considering Fe^2+^  + Fe^3+^) and the Mg/(Mg + Fe^2+^) ratio progressively decrease upon cooling at low *f*O_2_. The Mg/(Mg + Fe^2+^) ratio increases under oxidizing conditions (≥ NNO) at constant temperature similar to glass and olivine.

##### Calcium

Clinopyroxene CaO content remains constant upon cooling. It decreases from reducing conditions (≤ NNO-0.8) (ca. 21.0 wt%, ca. 0.85 apfu) to oxidizing conditions (≥ NNO) (ca. 21.0 to ca. 18.5 wt%, from 0.85 to 0.73 apfu), where the stability of plagioclase and pigeonite is increased. Wollastonite content (i.e. Ca_2_Si_2_O_6_ endmember) is ca. 0.40 at NNO-4 to NNO-0.8 and distinctly lower (ca. 0.35) from NNO to air, increasing slightly upon cooling. Decreasing CaO with increasing *f*O_2_ infers that silica activity increases with increasing *f*O_2_ and that clinopyroxene is approaching the two-pyroxene solvus (and finally reaching it in the case of coexisting pigeonite).

##### Chromium

According to Papike et al. (2016), within the *f*O_2_ range studied, all Cr occurs as Cr^3+^. Clinopyroxene Cr_2_O_3_ content depends strongly on temperature and *f*O_2_ (see Leuthold et al. 2015) and sector zoning. Cr_2_O_3_ concentration is very high (1.5 wt% at 1170 °C and NNO-4, bright sector; 1.2 wt% at 1170 °C and NNO-3, dark sector) under strongly reducing conditions at the point of saturation, dropping with cooling (0.4 wt% at 1110 °C and NNO-3, bright and dark sectors) and/or increased *f*O_2_ (0.04 wt% in air, from saturation temperature to solidus) (Fig. 3a).

Close to clinopyroxene saturation temperature, *D*_Cr_ is high (ca. 12–17, in dark and bright sectors respectively) under reducing conditions (NNO-4 to NNO-2) and thereafter decreases to ca. 3 in air, where ülvospinel (generally < 1.5 wt% Cr_2_O_3_) co-crystallizes and melt faction is lower (Online Resources 4 and 5). Under strongly reducing condition (NNO-9 to ca. NNO-2), Mallmann and O’Neill (2009) and Papike et al. (2016) showed the opposite trend, with increasing *D*_Cr_ from NNO-9 (i.e. where Cr occurs as Cr^2+^) to NNO-1 and constant *D*_Cr_ to NNO + 4. *D*_Cr_ is similar in picrite and basalt experiments at similar temperature and *f*O_2_, pointing to a minor effect of differentiation.

##### Vanadium

V concentration in clinopyroxene is strongly dependent on *f*O_2_ (Fig. 3e), with a progressive change of the V valence from V^3+^ (< NNO-3) to V^4+^ to V^5+^ (air) upon increasing *f*O_2_ (see Papike et al. 2016). At NNO-4, clinopyroxene V concentration varies from 2300 μg/g at 1170 °C to 1400 μg/g at 1125 °C. Under strongly oxidizing conditions (≥ NNO + 0.8), V is consistently low (100–300 μg/g) at 1170–1125 °C. *D*_V_ decreases strongly under oxidizing conditions, in agreement with Mallmann and O’Neill (2009), from ca. 10 at NNO-4 to ca. 0.2 in air but shows no correlation with temperature (except at NNO-4, where it increases upon cooling). V is not fractionated between bright and dark sectors. The V exchange mechanism appears more complex, as no clear correlation with Cr, Fe, Al or Ti is observed, possibly due to the variable valences.

##### Scandium

In contrast to vanadium, scandium has only one valence state (3 +) under the experimental conditions. Consequently, *D*_Sc_ is much less variable than *D*_V_. All *D*_Sc_ values lie between 2 and 6 (mean 3.49 ± 0.69) with no obvious correlation with crystal composition, temperature or *f*O_2_. Sc does not fractionate significantly between bright and dark sectors; enrichment can be seen in either sector but not by more than 20% relative.

##### Titanium and High Field Strength Elements

Clinopyroxene TiO_2_ concentration increases (ca. 1.0–2.4 wt%) upon cooling under reducing conditions (≤ NNO-0.8) but is constant under oxidizing conditions (ca. 1.3 wt%), where we approach saturation with an Fe-Ti-phase in the melt. TiO_2_ is enriched by a factor ca. 1.5 in bright sectors along a- and b-axes. At high temperature (≥ 1000 °C), *D*_Ti﻿_ only decreases slightly with temperature in bright sectors (from ca. 0.56 at 1175 °C to ca. 0.23 at 1110 °C) and exhibits none or little decrease (0.25–0.41) in dark sectors. It remains invariant with pressure, *f*O_2_ and melt chemistry (based on experimental databases of Hirschmann et al. 2008 [LEPR]; Bédard 2014; Villiger et al. 2007 [MORB at 0.7 and 1 GPa]; Skulski et al. 1994 [0.1–0.3 GPa basalt experiments]; Grove et al. 1992 [MORB experiments at 1 atm, 0.2 GPa and 0.8 GPa]; Gallahan and Nielsen 1992 [picrite and ankaramite one atmosphere experiments at QFM condition]; our basalt experiments [Leuthold et al. 2015; this study]). *D*_Ti﻿_ increases regularly with clinopyroxene Al^IV^ along a single *f*O_2_ buffer (i.e. Ti-Tschermak’s exchange), under reducing condition, in agreement with Wood and Trigila (2001). Ti/Al^IV^ varies from ca. 0.25 at NNO-4 to almost zero in air. Hammer (2006) showed the Ti/Al ratio increases under reducing condition, and at faster cooling rate. Our experiments (database of Leuthold et al. 2015) also show how starting material Ti/Al ratio plays an important role on these ratios. As for plagioclase, clinopyroxene *D*_﻿Ti_ appears well suited to calculate melt chemistry, although care is necessary in identifying the analysed face, as Ti shows appreciable sector zoning.

Titanium is the most abundant High Field Strength Element (HFSE) on the clinopyroxene M1 site (Hill et al. 2011) and serves as a proxy for other HFSE (Blundy and Wood 2003). We confirm observations by Forsythe et al. (1991), Skulski et al. (1994) and Shepherd et al. (2022) who reported linear correlations between *D*_Ti﻿_ and *D*_HFSE﻿_ values for clinopyroxene in basalts at 1 atm and 1–2.8 GPa. In our experiments, we see only subtle positive correlation between Al^IV^ and *D*_Zr_, *D*_Nb_ and *D*_Hf_, in contradiction with the strong increases described in Lundstrom et al. (1998) and Wood and Trigila (2001). There is no visible effect of *f*O_2_ on *D*_HFSE_. Tantalum concentration was too low in our experiments for robust discussion.

##### Rare Earth Elements and Yttrium

LREE (La to Gd) were measured precisely by LA-ICP-MS, whereas low-abundance HREE (Tb, Ho, Tm, Lu) show some significant scatter due to low (< 1 µg/g) concentrations. Clinopyroxene REE + Y increase by a factor of 2–3 upon cooling under reduced conditions but show little or no increase under oxidized conditions (Fig. 3f). *D*_REE+Y_ (Online Resource 6) lie in the range 0.04–0.86 decreasing, as expected, with higher ionic radius, from moderately incompatible Lu to Sm to strongly incompatible La. Using Sm as a representative REE, we see that *D*_Sm_ increases with decreasing temperature and from picrite to basalt starting compositions (Fig. 6a). There is no systematic effect of changing *f*O_2_. Cooling and fractionation processes thus have opposite effects on *D*_REE+Y_ such that the overall variation in *D* for the entire suite of clinopyroxenes is modest, e.g. 0.31–0.77 for *D*_Y_. There is no variation of *D*_REE+Y_ with NBO/T in the glass. In terms of crystal chemistry, *D*_Sm_ also increases with increasing Al^IV^ but not systematically (Fig. 6b). This variation is similar in both basalt and picrite experiments. There is a similarly scattered increase in D_Sm_ with increasing Fe^3+^ (not shown). Clinopyroxene Eu concentration is low, increasing from 0.3 µg/g at NNO-4 to 0.47 µg/g at ≥ NNO-0.7 and the Eu/Eu*^3^ is always < 1 in reduced experiments due to preferential incorporation of Eu^3+^ into clinopyroxene. Thus, clinopyroxene Eu/Eu* and *D*_Eu/Eu*_ are distinctly higher under reducing conditions, the latter increasing from ~ 0.6 at NNO-4 to 1.0 at NNO + 1 with a subordinate increase with decreasing temperature. Sector zoned clinopyroxene shows higher LREE + Y concentration in bright Al^IV^- and Fe^3+^-rich sectors (Fig. 6b), consistent with CaSi = REEAl^IV^ exchange. *D*_Eu_ increases under oxidized conditions in bright sectors but shows no clear variation in dark sectors. Our experiments do not go to sufficiently oxidised conditions to see any discernible effect on partitioning of Ce.

##### Large Ion Lithophile Elements

Strontium is the only LILE in clinopyroxene that was measured with sufficient precision to be considered. Sr concentration shows little variation in our experiments, between ca. 30 µg/g (≤ NNO-0.8) and ca. 40 µg/g (≥ NNO). *D*_Sr_ (~ 0.11) shows no clear variation with *f*O_2_ or temperature, due primarily to exchange with Ca, that itself shows little variation.

### Pigeonite

Toplis and Carroll (1995) showed that low-Ca clinopyroxene predicted by MELTS calculations under oxidizing conditions was absent in their experiments. In our experiments, pigeonite (8–12% wollastonite component) crystallizes (up to 15 vol%) under strongly oxidizing conditions from intermediate temperature (1140 °C at NNO + 0.7, ca. 1165 °C in air) down to the solidus (Fig. 1d and Online Resource 5). Its stabilization follows the olivine to pigeonite peritectic reaction in response to increased ratio of SiO_2_ to MgO + FeO in the melt (e.g. Longhi and Boudreau 1980) and melt polymerization (Fig. 2). However, we have no textural evidence for olivine to pyroxene reaction in our equilibrium experiments, and olivine and pigeonite appear to co-crystallize at ca. 1165 °C in air. The Fe^3+^/Fe_tot_ ratio in pigeonite (calculated using stoichiometry) increases strongly from 0.17 at NNO to 0.81 in air, with a constant FeO_t_ of ca. 5 wt% and low Cr_2_O_3_ (≤ 0.03 wt%, close to the limit of detection). Pigeonite crystals were too small for LA-ICP-MS analysis.

## Discussion

### Effect of *f*O_2_ on REE partitioning into clinopyroxene

**Fig. 7:**
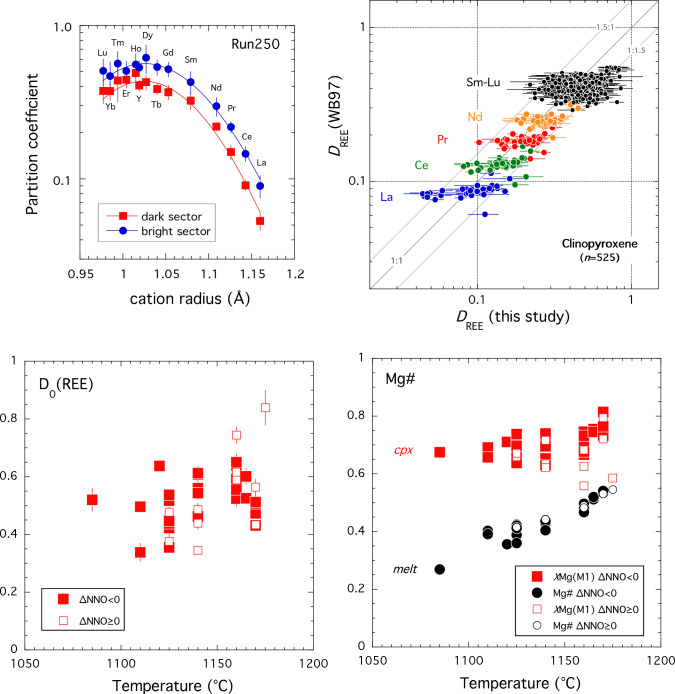
Onuma diagram for clinopyroxene-melt partition coefficients in sector-zoned crystal from run250 (1170 °C, NNO). Curves are separate least squares fits to the lattice strain model (Blundy and Wood 1994) for the bright and dark sectors; fit parameters in Online Resource 6. Note the higher *D*_REE_ in the bright sector, but overall similar patterns. *D*_Eu_ is not shown due to presence of both 3 + and 2 + valence state. Error bars are 1 s.d

The partitioning behaviour of REE + Y in terms of ionic radii (in VIII-fold co-ordination; Shannon 1976) can be described well by the lattice strain model of Blundy and Wood (1994) notwithstanding scatter for *D*_HREE_ from some runs resulting from analytical uncertainty. To explore the effects of *f*O_2_ on REE partitioning we have fitted the lattice strain model to 40 experiments in which *D*_REE+Y_ is precisely determined, including nine runs with sector zoned crystals. Typical fits for a sector-zoned clinopyroxene from run250 are shown in Fig. 7. Clinopyroxene-melt *D*_REE_ and lattice strain fit parameters (*r*_0_, *E*, *D*_0_) were obtained for all runs using a weighted least squares regression and are reported in Online Resource 6. For sector zoned pyroxenes *D*_0_ is consistently higher in bright sector (typically by 3–32% relative); *r*_0_ can be both larger (by up to 0.016 Å) or smaller (< 0.017 Å) in the bright sector. *E* is the same within error for both sectors. Thus, both sectors tend to describe similar, sub-parallel parabolae (Fig. 7).

**Fig. 8:**
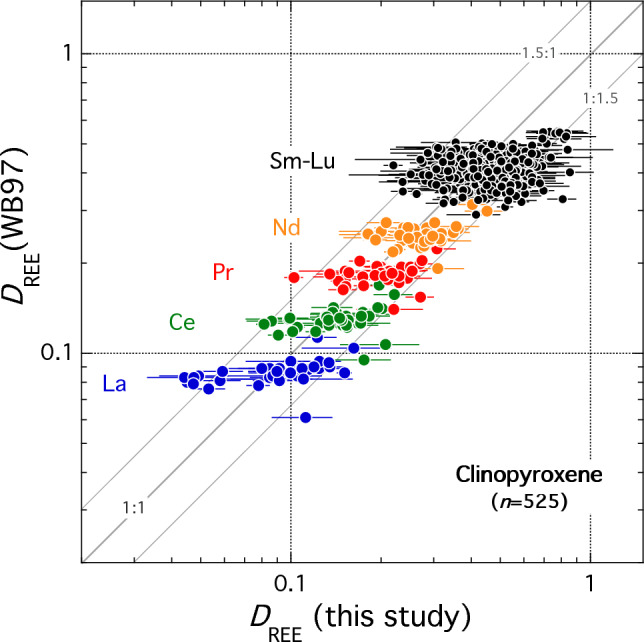
Comparison of calculated (Wood and Blundy 1997) versus experimental *D*_REE_ from this study. Calculations were performed using the experimental crystal and melt composition assuming all Fe as Fe^2+^ in both phases and the experimental temperature with the REEMgAlSiO_6_ model. Error bars on experimental data are 1 s.d. The three parallel lines denote 1:1, 1.5:1 and 1:1.5 correlations. For the total 525 individual *D*_REE_ determinations, with few exceptions calculated *D*_REE_ lie within a factor of ± 1.5 of the experimental values. *D*_Eu_ is not plotted

Fit parameters are in good agreement with those predicted using the MgREEAlSiO_6_ partitioning model of Wood and Blundy (1997): average absolute deviations are 19% relative in *D*_0_, 0.006 Å in *r*_0_ and 53 GPa in *E*. For the entire dataset, *D*_REE_ calculated using the Wood and Blundy (1997) REEMgAlSiO_6_ model (taking all Fe as Fe^2+^ in both clinopyroxene and melt) lies within 1 s.d. of the measured *D*s for all REE for all but 7 determinations out of a total of 525 individual *D*_REE_ (Fig. 8). This is well within the expected accuracy of the Wood and Blundy (1997) model despite the fact that the present experiments lie outside the original calibration dataset. We note that using stoichiometry to estimate Fe^3+^ in clinopyroxene and Kress and Carmichael (1991) to estimate Fe^3+^ in melt does not significantly change the quality of the model predictions due to competing effects on melt Mg# and clinopyroxene M1-site occupancy.

**Fig. 9:**
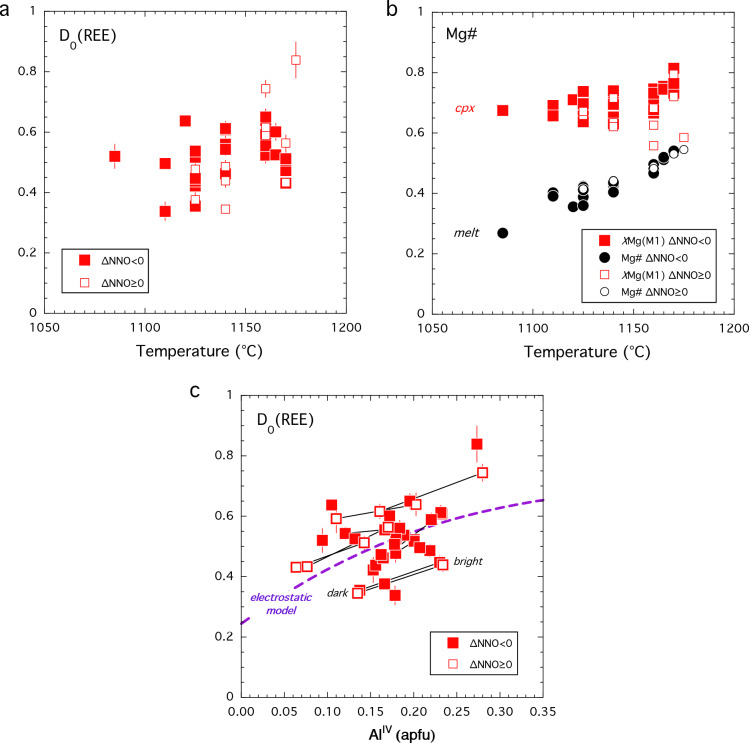
Clinopyroxene-melt partitioning of REE. In all plots filled symbols denote experiments at ∆NNO < 0; open symbols ∆NNO ≥ 0. **a** *D*_0_ versus temperature. **b** Mg# versus temperature. Red symbols denote the Mg occupancy of the M1-site; black symbols denote Mg# of the coexisting melt. Note latter parameters decreases more rapidly with decreasing temperature than the former accounting for temperature dependence observed in (**a**). Calculations assume all Fe as Fe^2+^; the overall variation is the same if estimates of Fe^3+^ in both phases are used for calculations. **c** *D*_0_ versus Al^IV^. For sector zoned clinopyroxenes the thin black lines connect bright and dark sectors; the latter always lies at lower Al^IV^ than the former. The purple dashed line shows the electrostatic model of Wood and Blundy (2001) calculated at 1150 °C for clinopyroxenes along the diopside-CaTs or diopside-FATs joins. Note that bright-dark tie lines parallel the model predications indicating the REE fractionation between sectors reflects availability of suitably charged M2 sites for REE^3+^ occupancy

In terms of temperature, *D*_0_ decreases slightly with decreasing temperature (Fig. 9a) from ~ 0.8 to ~ 0.3 due to the competing effects of temperature and differentiation noted above. There is no discernible difference between oxidised and reduced experiments in this plot (Fig. 9a). In terms of the Wood and Blundy (1997) REEMgAlSiO_6_ model, the temperature effect can be explained because the Mg# of the melt decreases more rapidly than the Mg occupancy of the M1-site in our experimental suite (Fig. 9b). These two parameters work in opposition to drive *D*_0_ down despite the fact that, at constant composition and pressure, *D*_0_ is predicted to increase from 0.24 to 0.38 with decreasing temperature from 1175 to 1085 °C (Wood and Blundy 1997).

*D*_0_ is weakly correlated with Al^IV^ (Fig. 9c) and to a lesser extent with Fe^3+^ calculated from stoichiometry (not shown). Wood and Blundy (2001) show that the dependence of *D*_0_ on crystal composition can be usefully considered in terms of the availability of suitably charged sites in the clinopyroxene lattice and the electrostatic energy penalty associated with placing an REE^3+^ ion onto a site with inappropriate charge. In detail, the availability of suitably charged sites depends on the exact crystal composition taking into account all cation site occupancies. In Fig. 9c we show the predicted behaviour along the diopside-CaTs (and diopside-FATs) binary joins at a temperature of 1150 °C using the same electrostatic energy term (∆*G*_elec_ = 28 kJ/mol) as proposed by Wood and Blundy (2001) for ‘low-Al_2_O_3_ pyroxene’; for higher Al_2_O_3_ pyroxenes ∆*G*_elec_ decreases to ~ 19 kJ/mol. Following Wood and Blundy (2001) the zero-Al^IV^ intercept is pinned at a notional value, in this case 0.24. Figure 9c shows that the overall variation in *D*_0_ is consistent with the electrostatic theory of Wood and Blundy (2001). The scatter in the plot reflects the fact that the data are not truly isothermal, i.e., the zero-Al^IV^ intercept will vary with temperature, the presence of additional cations in the lattice that are not present on Di-CaTs or Di-FATs joins, and the crystal-chemical dependence of ∆*G*_elec_. Significantly, however, where we have data for coexisting sectors in a single clinopyroxene we see that the tie-line connecting the two parallels the electrostatic model curves consistently. There is no difference in behaviour between sector zoned clinopyroxenes grown under oxidised versus reduced conditions consistent with the similar influence of M1-site Fe^3+^ and Al^VI^ on the overall distribution of cation site charges. Thus, we suggest that REE variation between adjacent sectors of pyroxene is controlled entirely by electrostatic effects namely the availability of suitably charged sites and electrostatic energy penalty for charge-mismatched sites.

We conclude that *f*O_2_ has limited effect of the partitioning of REE (except for polyvalent Eu) into clinopyroxene. The dominant influence on *D*_0_ in our dataset is the Mg# of the melt and the Mg occupancy of the M1-site. Although both parameters are affected by changing *f*O_2_, the effect is adequately captured by the predictive model of Wood and Blundy (1997). The presence of Fe^3+^ on M1 sites is broadly similar to that of Al^3+^ for FATs- and CaTs-type substitutions, respectively, such that electrostatic effects on *D*_0_ are similar under high and low *f*O_2_ as evidenced by sector-zoned grains (Fig. 9c). Eu is the only REE studied here that is affected by *f*O_2_; our experiments do not go to sufficiently oxidised conditions to see any discernible effect on partitioning of Ce.

### Trace element oxybarometry

Elements with multiple valences under magmatic conditions (Fe, Cr, V, Eu) are strongly affected by *f*O_2_. Consequently, there is long-standing interest in using the mineral-melt partitioning of multivalent cations as oxybarometers (Mallmann et al. 2021). Fe is a major element in olivine (all Fe^2+^), spinel and clinopyroxene and a minor element in plagioclase. Cr and V partition into spinel, as well as clinopyroxene. Eu^2+^ partitions strongly into plagioclase. Our picritic-basaltic system was saturated with olivine, spinel, plagioclase and clinopyroxene in most experiments across a wide range in *f*O_2_, therefore it is instructive to assess the potential of element partitioning into these phases as oxybarometers.

We do not consider further Fe in olivine or pyroxenes because it is a major cation in these minerals and the estimation of Fe^3+^/Fe^2+^ via stoichiometry is insufficiently precise. Fe^3+^ is excluded from the olivine structure, thus *D*_Fe_ for olivine is sensitive primarily to the Fe^3+^ content of the melt. The effect of redox on olivine-melt partitioning of Fe has been discussed recently by Blundy et al (2020) and is not revisited here. The behaviour of Fe^3+^ in clinopyroxene is further complicated by alternative possible substitution mechanisms (acmite, FATs). Fe is a trace element in plagioclase, however, and more readily incorporated as Fe^3+^ than Fe^2+^ (Phinney 1992). Consequently, plagioclase FeO_tot_ content increases under oxidizing conditions with very limited effect of temperature (Fig. 4). Pressure also has a strong effect on plagioclase/glass *D*_Fe_ (Wilke and Behrens 1999). Using France et al. (2010) model for FeO_tot_ in plagioclase, we obtain a strong correlation between experimental and calculated *f*O_2_ (*R* = 0.75), even under reducing conditions. However, at our experimental conditions, *f*O_2_ is over-estimated by ca. 3 log units (∆NNO_calc_ = 0.6·∆NNO_exp_ + 3). Caution is therefore necessary with FeO in plagioclase oxybarometers.

Our results reveal that clinopyroxene Cr_2_O_3_ concentrations and *D*_Cr_ in the picritic system are strongly dependent on *f*O_2_ (Fig. 3a,d). However, temperature also strongly affects clinopyroxene Cr_2_O_3_ concentration. For elements fractionated between different sectors, extra uncertainties are added when natural grain faces are not characterised. Cr concentrations in olivine and spinel strongly decrease under oxidised conditions but also upon cooling and crystallization. Consequently, it is not advised to employ Cr concentrations and partitioning as an oxybarometer. Fe and Cr in spinel are affected by a wide range of differentiation processes (e.g. Leuthold et al. 2015) and are not readily formulated as oxybarometers.

We conclude that the only trace elements best suited to use as oxybarometers are V (in olivine and clinopyroxene) and Eu (in plagioclase). In the following, we develop the use of olivine and clinopyroxene *D*_V_ and plagioclase *D*_Eu_ as oxybarometers for basaltic systems by building, respectively, on the work of Mallmann and O’Neill 2009, 2013) and Aigner-Torres et al (2007).

#### Theoretical background

Homogenous equilibrium between species of different charge in silicate melts is conveniently described by the redox potential, E’, defined as log_10_ of the equilibrium constant for the relevant redox reactions (Schreiber 1987), which for Eu and V are:

1 $$E^{\prime}_{Eu2/3} = \frac{1}{4}{\text{log}}_{10} fO_2 + {\text{log}}_{10} \left( {\frac{{{\text{Eu}}^{2 + } }}{{{\text{Eu}}^{3 + } }}} \right)$$

2a $$E^{\prime}_{V2/5} = \frac{3}{4}{\text{log}}_{10} fO_2 + {\text{log}}_{10} \left( {\frac{{V^{2 + } }}{{V^{5 + } }}} \right)$$

2b $$E^{\prime}_{V3/5} = \frac{2}{4}{\text{log}}_{10} fO_2 + {\text{log}}_{10} \left( {\frac{{V^{3 + } }}{{V^{5 + } }}} \right)$$

2c $$E^{\prime}_{V4/5} = \frac{1}{4}{\text{log}}_{10} fO_2 + {\text{log}}_{10} \left( {\frac{{V^{4 + } }}{{V^{5 + } }}} \right)$$

Note that for V we use redox couples between V^5+^ and more reduced states as this simplifies the expressions for partition coefficients (Mallmann and O’Neill 2009). Values of *E*′ vary with both melt composition and temperature. To remove the dependence on the latter we will define *f*O_2_ in log_10_ units relative to the NNO buffer (∆NNO) at the pressure and temperature of interest, as formulated by O'Neill and Pownceby (1993), to create variants on *E*′ that we designate E^*^:

3 $$E_{Eu2/3}^* = \frac{1}{4}\Delta {\text{NNO}} + {\text{log}}_{10} \left( {\frac{{{\text{Eu}}^{2 + } }}{{{\text{Eu}}^{3 + } }}} \right)$$

4a $$E_{V2/5}^* = \frac{3}{4}\Delta {\text{NNO}} + {\text{log}}_{10} \left( {\frac{{V^{2 + } }}{{V^{5 + } }}} \right)$$

4b $$E_{V3/5}^* = \frac{2}{4}\Delta {\text{NNO}} + {\text{log}}_{10} \left( {\frac{{V^{3 + } }}{{V^{5 + } }}} \right)$$

4c $$E_{V4/5}^* = \frac{1}{4}\Delta {\text{NNO}} + {\text{log}}_{10} \left( {\frac{{V^{4 + } }}{{V^{5 + } }}} \right)$$

We can then write the partition coefficient for Eu in terms of three variables $D_{{\text{Eu}}^{3 + } }$, $D_{{\text{Eu}}^{2 + } }$ and $E_{Eu2/3}^*$, as follows (cf. Aigner-Torres et al. 2007):

5a $$D_{{\text{Eu}}} = \frac{{D_{{\text{Eu}}^{3 + } } + D_{{\text{Eu}}^{2 + } } \times 10^{\left( {E_{Eu2/3}^* - \frac{1}{4}\Delta {\text{NNO}}} \right)} }}{{1 + 10^{\left( {E_{Eu2/3}^* - \frac{1}{4}\Delta {\text{NNO}}} \right)} }}$$

For vanadium in clinopyroxene and olivine, the expression for *D*_V_ is more complicated due to multiple oxidation states (cf. Mallmann and O’Neill 2009), giving rise to seven independent variables, $D_{V^{5 + } }$, $D_{V^{4 + } }$, $D_{V^{3 + } }$, $D_{V^{2 + } }$, $E_{V2/5}^*$, $E_{V3/5}^*$ and $E_{V4/5}^*$:

6a $$D_V = \frac{{D_{V^{5 + } } + D_{V^{4 + } } \times 10^{\left( {E_{V4/5}^* - \frac{1}{4}\Delta {\text{NNO}}} \right)} + D_{V^{3 + } } \times 10^{\left( {E_{V3/5}^* - \frac{2}{4}\Delta {\text{NNO}}} \right)} + D_{V^{2 + } } \times 10^{\left( {E_{V2/5}^* - \frac{3}{4}\Delta {\text{NNO}}} \right)} }}{{1 + 10^{\left( {E_{V4/5}^* - \frac{1}{4}\Delta {\text{NNO}}} \right)} + 10^{\left( {E_{V3/5}^* - \frac{2}{4}\Delta {\text{NNO}}} \right)} + 10^{\left( {E_{V2/5}^* - \frac{3}{4}\Delta {\text{NNO}}} \right)} }}$$

Equations (5a) and (6a) can then be fitted to partitioning data to obtain the independent parameters by least-squares regression.

Using partition coefficients for single, redox-sensitive trace elements as oxybarometers can be complicated by the fact that the effects of redox, crystal composition and temperature may be conflated. For example, variation in *D*_V_ may arise because of both changes in its valence state and changes in the lattice site parameters that control 2+ and 3+ cation substitution, such as Al or Ca content or Mg#. In the case of Eu partitioning, the strong anorthite dependence of *D*_Sr_ (e.g. Blundy and Wood 1991; Dohmen and Blundy 2014), which is similar in size to Eu^2+^, confers variability in *D*_Eu_ that is unrelated to *f*O_2_. For these reasons it can be useful to adapt Eqs. (5a) and (6a) by referencing the polyvalent cation to another, compatible cation that is similar in charge and size to one of the valence states considered. Thus, for Eu in plagioclase, we ratio *D*_Eu_ to *D*_Sr_. The resulting expression becomes (cf. Aigner-Torres et al. 2007):

5b $$\frac{{D_{{\text{Eu}}} }}{{D_{{\text{Sr}}} }} = \frac{{\frac{{D_{{\text{Eu}}^{3 + } } }}{{D_{{\text{Sr}}} }} + \frac{{D_{{\text{Eu}}^{2 + } } }}{{D_{{\text{Sr}}} }} \times 10^{\left( {E_{Eu2/3}^* - \frac{1}{4}\Delta {\text{NNO}}} \right)} }}{{1 + 10^{\left( {E_{Eu2/3}^* - \frac{1}{4}\Delta {\text{NNO}}} \right)} }}$$

The situation for V is more complex because of its four possible valence states (V^2+^, V^3+^, V^4+^, V^5+^) in the *f*O_2_ range considered meaning that a single reference cation cannot be easily chosen. The closest matches in ionic radius (Shannon 1976) are: Zn^2+^ (0.74 vs. 0.79 Å for V^2+^); Ga^3+^ (0.62 vs. 0.64 Å); Ti^4+^ (0.605 vs. 0.58 Å); Nb^5+^ (0.64 vs. 0.54 Å). Of these possibilities, Ga would have the greatest potential as a normalising species over the range of terrestrial *f*O_2_ where V^3+^ is the most abundant species. However, the low abundance of Ga in our experiments leads to uncertainties on the *D*_V_/*D*_Ga_ ratio of around 35% relative. We have therefore chosen Sc for normalisation. Although the ionic radius compared to V^3+^ is sub-optimally large (V^3+^ = 0.640 Å; Sc^3+^  = 0.745 Å), this element pair has the advantage of precise experimental determination (mean relative error on *D*_V_/*D*_Sc_ = 19%) and has been used previously (e.g. Mallmann and O’Neill 2013; Wang et al. 2019). Moreover, *D*_Sc_ is typically independent of temperature, *f*O_2_ and crystal composition in other experimental series on basalts, e.g. 1.51 ± 0.13 (Mallmann and O’Neill 2009), 4.97 ± 0.45 (Shepherd et al. 2022), 1.23 ± 0.16 (Karner et al. 2008). The expression for D_V_/D_Sc_, adapted from Eq. (6a), becomes:

6b $$\frac{D_V }{{D_{{\text{Sc}}} }} = \frac{{\frac{{D_{V^{5 + } } }}{{D_{{\text{Sc}}} }} + \frac{{D_{V^{4 + } } }}{{D_{{\text{Sc}}} }} \times 10^{\left( {E_{V4/5}^* - \frac{1}{4}\Delta {\text{NNO}}} \right)} + \frac{{D_{V^{3 + } } }}{{D_{{\text{Sc}}} }} \times 10^{\left( {E_{V3/5}^* - \frac{2}{4}\Delta {\text{NNO}}} \right)} + \frac{{D_{V^{2 + } } }}{{D_{{\text{Sc}}} }} \times 10^{\left( {E_{V2/5}^* - \frac{3}{4}\Delta {\text{NNO}}} \right)} }}{{1 + 10^{\left( {E_{V4/5}^* - \frac{1}{4}\Delta {\text{NNO}}} \right)} + 10^{\left( {E_{V3/5}^* - \frac{2}{4}\Delta {\text{NNO}}} \right)} + 10^{\left( {E_{V2/5}^* - \frac{3}{4}\Delta {\text{NNO}}} \right)} }}$$

##### Vanadium in olivine

**Fig. 10:**
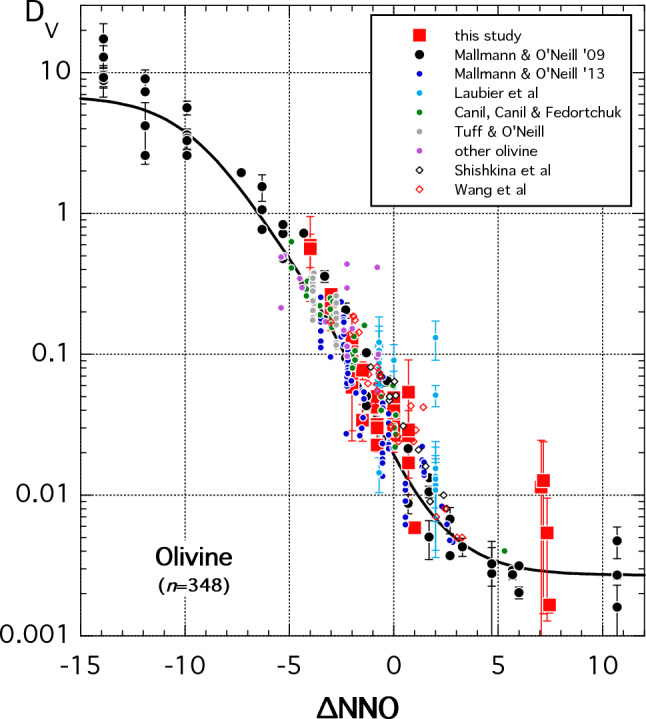
Vanadium partitioning into olivine as a function of *f*O_2_ expressed in log units relative to relative to NNO (∆NNO). Solid line is a fit of Eq. (6a) to the entire dataset using vanadium redox potentials calculated from Mallmann and O’Neill (2009) and the method described in text and parameters listed in Table 2. Error bars are 1 s.d. Data sources in addition to this study are: Canil (1997, 1999), Canil and Fedortchouk (2001), Herd et al. (2002), Shearer et al. (2006), Mallmann and O’Neill (2009, 2013), Tuff and O’Neill (2010), Papike et al. (2013), Davis et al. (2017), Laubier et al. (2014), Shishkina et al. (2018), Wang et al. (2019) and Dygert et al. (2020)

In Fig. 10 we plot olivine *D*_V_ for our experiments alongside data from the literature (Canil 1997, 1999; Canil and Fedortchouk 2001; Herd et al 2002; Shearer et al. 2006; Mallmann and O’Neill 2009, 2013; Tuff and O’Neill 2010; Papike et al. 2013; Davis et al. 2017; Laubier et al 2014; Shishkina et al. 2018; Wang et al. 2019; Dygert et al. 2020). We observe that all of the data describe a single curve with limited scatter despite the wide range in temperature, pressure, melt composition and olivine composition. Normalising by *D*_Sc_ (not shown) increases the scatter, so we use Eq. (6a) for fitting purposes. A global, weighted fit of the entire (*n* = 348) dataset to Eq. (6a) was performed using the values $E_{V2/5}^*$, $E_{V3/5}^*$ and $E_{V4/5}^*$ from Mallmann and O’Neill (2009). These were converted from their homogeneous equilibrium constants, $K^{\prime}_{\hom }$, to ∆NNO at 1 bar and 1300 °C (the conditions of their experiments) simply by taking into account log_10_*f*O_2_ of NNO at 1300 °C, i.e. –6.689. Thus,

7a $$E_{V2/5}^* = \frac{3}{4} \times 6.689 - {\text{log}}_{10} K^{\prime}_{\hom \left( {6a} \right)} = - 3.587$$

7b $$E_{V3/5}^* = \frac{2}{4} \times 6.689 - {\text{log}}_{10} K^{\prime}_{\hom \left( {6b} \right)} = - 1.174$$

7c $$E_{V4/5}^* = \frac{1}{4} \times 6.689 - {\text{log}}_{10} K^{\prime}_{\hom \left( {6c} \right)} = - 0.838$$

where subscripts *hom(6a)*, *hom(6b)* and *hom(6c)* are those used by Mallmann and O’Neill (2009) to describe the homogeneous equilibria involving V^5+^–V^2+^, V^5+^–V^3+^ and V^5+^–V^4+^ respectively. Our fit to olivine *D*_V_ yields the parameters given in Table 2. Note that these fit parameters are very close to those of Mallmann and O’Neill (2009) because of the considerable span of *f*O_2_ that they cover compared to the rest of the fitted data. The fitted expression, which contains no temperature, pressure or compositional terms, reproduces *D*_V_ for the entire calibration dataset with an average relative deviation of 35.6% across a pressure–temperature range of 0.001–30 kbar and 1025–1530 °C. For comparison, the expression of Mallmann and O’Neill (2013), containing four discrete compositional terms, reproduces *D*_V_ for a smaller (*n* = 175), 1 bar dataset with an average relative deviation of 16%. Thus, the expression presented here is useful in situations where the melt composition is not known a priori, for example fractional melting or crystallisation calculations. Equation (6a) is not easily rearranged in terms of ∆NNO. For recovery of *f*O_2_ from olivine-melt *D*_V_, the composition-sensitive expression of Mallmann and O’Neill (2013) or the composition-independent expression of Shishkina et al (2018) are recommended. These two expressions reproduce our new experimental partitioning data (*n* = 41) with average absolute deviations of ± 0.64 and ± 0.77 log units, respectively, in the *f*O_2_ range NNO-4 to NNO + 2 confirming the potential of olivine-melt *D*_V_ as a precise and accurate oxybarometer.

**Table 2:** Fit parameters for D(V), D(V)/D(Sc) and D(Eu)/D(Sr)

Mineral	Parameter	Value	SD	Source
Olivine and clinopyroxene	E^*^(V^2+^–V^5+^)	− 3.587	0.059	1
	E^*^(V^3+^–V^5+^)	− 1.174	0.037	1
	E^*^(V^4+^–V^5+^)	− 0.838	0.123	1
Clinopyroxene	D(V^2+^)/D(Sc)	0.228	0.024	2
	D(V^3+^)/D(Sc)	2.66	0.05	2
	D(V^4+^)/D(Sc)	0.286	0.033	2
	D(V^5+^)/D(Sc)	0.0134	0.0006	2
Clinopyroxene^a^	D(V^2+^)	0.764	0.059	2
	D(V^3+^)	4.642	0.058	2
	D(V^4+^)	0.607	0.046	2
	D(V^5+^)	0.0169	0.0006	2
Olivine^a^	D(V^2+^)	6.84	0.05	2
	D(V^3+^)	0.107	0.002	2
	D(V^4+^)	0.0809	0.0016	2
	D(V^5+^)	0.00270	0.00004	2
Plagioclase	E^*^(Eu^2+^–Eu^3+^)	− 1.463	0.004	2
	D(Eu^2+^)/D(Sr)	1.047	–	3
	D(Eu^3+^)/D(Sr)	0.0080	0.0023	2

Sources: (1) Mallmann and O’Neill (2009), (2) This study and (3) Dohmen and Blundy (2014)

^a^Fit parameters differ slightly from those in Table 6 of Mallmann and O’Neill (2009) that were based on subsets of their experiments. All of their data were fitted simultaneously in this study

#### Vanadium in clinopyroxene

**Fig. 11:**
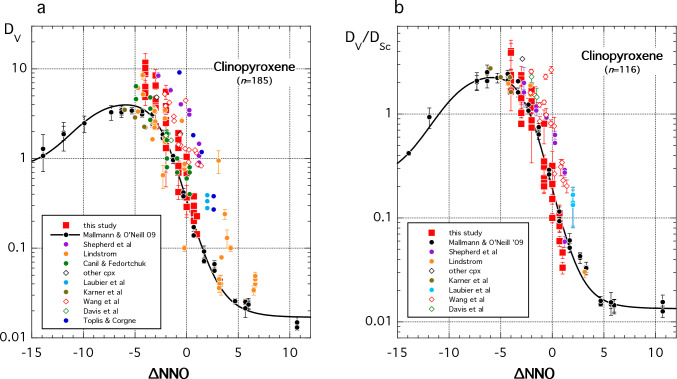
Vanadium partitioning into clinopyroxene as a function of ∆NNO. **a** *D*_V_. Solid line shows fit of Eq. (6) to the entire dataset of Mallmann and O’Neill (2009) using their redox potentials; fit parameters given in Table 2. Other datasets are variably offset from this fit, typically to higher *D*_V_. **b** *D*_V_/*D*_Sc_ showing significant reduction in vertical scatter of the data compared to (**a**). Solid line shows fit of Eq. (6b) to all data using Mallmann and O’Neill (2009) redox potentials; fit parameters given in Table 2. Data sources in addition to this study, with conditions ranging from 0.001 to 30 kbar and 1080–1470 °C, are: Lindstrom 1976; Jenner et al. 1993; Canil and Fedortchouk 2000; Pertermann and Hirschmann 2002; Toplis and Corgne 2002; Karner et al. 2008; Mallmann and O’Neill 2009; Davis et al. 2017; Laubier et al 2014; Wang et al. 2019; Shepherd et al. 2022. Data not labelled individually are included in ‘other cpx’. Not all studies report *D*_Sc_ hence fewer data are plotted in (**b**). Error bars for representative studies are 1 s.d

In Fig. 11a we plot clinopyroxene *D*_V_ for our experiments alongside data from the literature (Lindstrom 1976; Jenner et al. 1993; Canil and Fedortchouk 2000; Pertermann and Hirschmann 2002; Toplis and Corgne 2002; Karner et al. 2008; Mallmann and O’Neill 2009; Davis et al. 2017; Laubier et al 2014; Wang et al. 2019; Shepherd et al. 2022). The data (*n* = 185) span a wide range of pressure, temperature and crystal and melt composition. They show consistent behaviour in terms of *f*O_2_ in the interval NNO-5 to NNO + 7 although the data are spread over almost an order of magnitude in *D*_V_ at a given *f*O_2_. There is a general trend to higher *D*_V_ at lower temperature and higher pressure, but this behaviour is not systematic. There are too few data below NNO-5 to assess if the data spread persists to very reducing conditions. Much of the spread in Fig. 11a can be eliminated by plotting *D*_V_ normalised to *D*_Sc_ (Fig. 11b). We have therefore fitted *D*_V_/*D*_Sc_ to Eq. (6b) using a weighted least squares routine and the same values of $E_{V2/5}^*$, $E_{V3/5}^*$ and $E_{V4/5}^*$ as for olivine. The parameter values are given in Table 2. The fitted expression, which contains no temperature, pressure or compositional terms, reproduces *D*_V_/*D*_Sc_ for the entire calibration dataset (*n* = 116) with an average relative deviation of 35.3% across a pressure–temperature range of 0.001–30 kbar and 1080–1470 °C. A tendency for higher *D*_V_/*D*_Sc_ at higher pressures (data of Davis et al. 2017 and Wang et al. 2019) remains. Although we have not attempted to express ∆NNO as a function of *D*_V_/*D*_Sc_, it is apparent that the spread in this ratio at a given *f*O_2_ (Fig. 11b) remains too great for clinopyroxene *D*_V_/*D*_Sc_ to be used as a precise oxybarometer. Moreover, the curvature of the variation with ∆NNO means that solutions are not unique in the range NNO-15 to NNO-2. Nonetheless, clinopyroxene Sc/V ratios can be used to provide qualitative *f*O_2_ information.

#### Europium in plagioclase

**Fig. 12:**
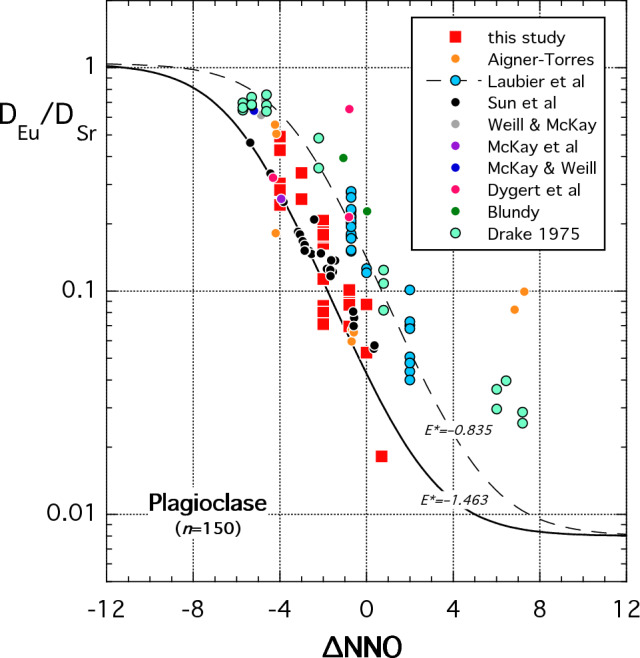
Europium partitioning into plagioclase, expressed as *D*_Eu_/*D*_Sr_, as a function ∆NNO. Data sources in addition to this study are: Sun et al. (1974), Drake (1975), Weill and McKay (1975), McKay and Weill (1977), McKay et al. (1994), Blundy (1997), Aigner-Torres et al. (2007), Laubier et al. (2014) and Dygert et al. (2020). Solid black line is fit to Eq. (5b) with fixed $\frac{{D_{{\text{Eu}}^{2 + } } }}{{D_{{\text{Sr}}} }}$ of 1.047 (see text for details) for the data from this study and from Sun et al. (1974) only. Fit parameters are given in Table 2. For comparison the dashed black line shows a fit to the data of Laubier et al (2014) using the same values of $\frac{{D_{{\text{Eu}}^{2 + } } }}{{D_{{\text{Sr}}} }}$ and $\frac{{D_{{\text{Eu}}^{3 + } } }}{{D_{{\text{Sr}}} }}$ as in Table 2. The fit value of $E_{Eu2/3}^*$ = –0.835 ± 0.023 indicates the sensitivity of *D*_Eu_/*D*_Sr_ to melt composition

In Fig. 12 we plot *D*_Eu_/*D*_Sr_ for our experiments together with published 1 atmosphere experimental data for natural basaltic compositions (Sun et al. 1974; Drake 1975; Weill and McKay 1975; McKay and Weill 1977; McKay et al. 1994; Blundy 1997; Aigner-Torres et al. 2007; Laubier et al. 2014; Dygert et al. 2020) covering a temperature range of 1100–1220 °C. Because of the influence of plagioclase composition on partition coefficients (Blundy and Wood 1991), normalisation to *D*_Sr_ is a useful first step to reduce scatter apparent in *D*_Eu_ from the different studies. Nonethelesss, considerable scatter persists despite the relatively limited range in temperature. Fitting *D*_Eu_/*D*_Sr_ to Eq. (5a) requires estimates of $\frac{{D_{{\text{Eu}}^{3 + } } }}{{D_{{\text{Sr}}} }}$ and $\frac{{D_{{\text{Eu}}^{2 + } } }}{{D_{{\text{Sr}}} }}$. Both can be readily obtained using the lattice strain model of Blundy and Wood (1994). $\frac{{D_{{\text{Eu}}^{2 + } } }}{{D_{{\text{Sr}}} }}$ is approximately 1, due to the close ionic radii of Eu^2+^ and Sr^2+^. From the lattice strain parameters of Dohmen and Blundy (2014) we calculate $\frac{{D_{{\text{Eu}}^{2 + } } }}{{D_{{\text{Sr}}} }}$ = 1.047 (Table 2). $\frac{{D_{{\text{Eu}}^{3 + } } }}{{D_{{\text{Sr}}} }}$ can be obtained by interpolation between *D*_Sm_ and *D*_Gd_ where these data are available (e.g. Dygert et al. 2020) or from a lattice strain fit to more sparse *D*_REE_ data. We apply the latter approach to our new experimental data as well as the datasets of Laubier et al. (2014) and Aigner-Torres et al. (2007). In all cases we only include experiments where the *D*_REE_ describe parabolic trends. These data were fitted using the lattice strain *r*_0_ values calculated for the relevant plagioclase An content using the expression of Dohmen and Blundy (2014). The calculated values of $\frac{{D_{{\text{Eu}}^{3 + } } }}{{D_{{\text{Sr}}} }}$ for all experiments lie in the range 0.004–0.016; the global average of 29 experiments is 0.0080 ± 0.0023 (Table 2) in good agreement with a value of 0.0074 calculated using the lattice strain data in Dohmen and Blundy (2014) for temperatures and plagioclase compositions similar to those in the experiments. The data were then fitted to Eq. (5a) using these values, which define the asymptotes of the observed variation. A fit to the present data and the Sun et al (1974) data yields $E_{Eu2/3}^*$ = –1.463 ± 0.004 (Fig. 12). Evidently, this fit does not reproduce the Laubier et al. (2014) data, which require a less negative value of $E_{Eu2/3}^*$ (–0.835 ± 0.023). The change in $E_{Eu2/3}^*$ confirms the sensitivity of Eu redox potential to melt composition, as discussed by Aigner-Torres et al. (2007). It is unlikely that the sensitivity of $E_{Eu2/3}^*$ to melt composition can explain the unusually high D_Eu_/D_Sr_ at high *f*O_2_ observed by Drake (1975) and Aigner-Torres et al (2007). In the case of Drake (1975) the low Eu content of plagioclases equilibrated in air is the likely cause, as noted by the author. Plagioclases synthesised at lower *f*O_2_, where Eu contents are higher, lie close to the Laubier et al. (2014) fit. The discrepancy of the oxidised Aigner-Torres et al (2007) data is less clear, although we note that the *D*_REE_ patterns for these experiments are not easily fit to the lattice strain model, suggestive of an analytical issue. The compositional sensitivity of $E_{Eu2/3}^*$ precludes the use of *D*_Eu_/*D*_Sr_ as a reliable oxybarometer, although for broadly basaltic systems the expression in Table 2 gives a good description of the variation of *D*_Eu_/*D*_Sr_ with *f*O_2_.

### Vanadium redox speciation

**Fig. 13:**
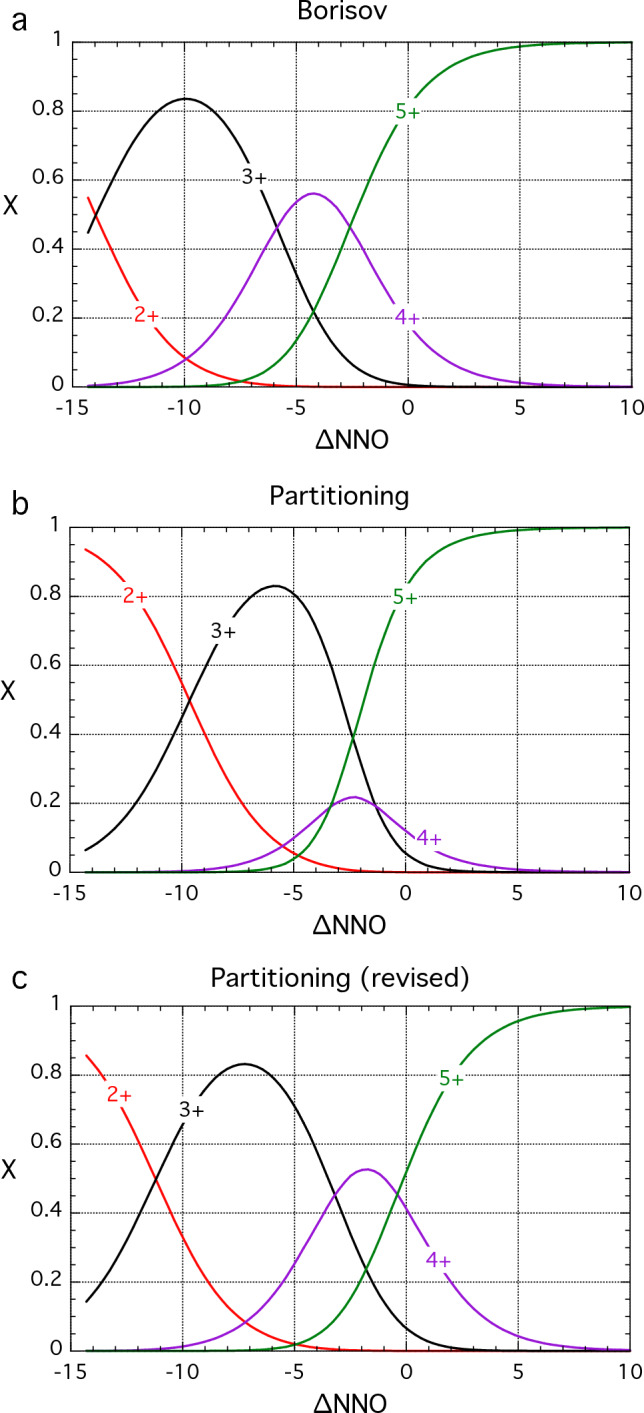
Vanadium redox speciation in silicate melts calculated using redox potentials from **a** Borisov (2013) at 1300 °C; **b** Mallmann and O’Neill (2009); and **c** extreme-fit values consistent with trace element partitioning (see text for details). All redox potentials given in Table 2

Our study of vanadium partitioning as a function of *f*O_2_ provides insights into redox speciation of *V* in silicate melts. Although the values of $E_{V2/5}^*$, $E_{V3/5}^*$ and $E_{V4/5}^*$ in Table 2 were obtained by Mallmann and O’Neill (2009) by fitting their partitioning data, they should be comparable to independent measurement of V speciation, for example from spectroscopy. For sodium disilicate melt $E_{V2/5}^*$, $E_{V3/5}^*$ and $E_{V4/5}^*$ as a function of temperature are provided by Borisov (2013). In Fig. 13a we plot the speciation as a function of ∆NNO using his values calculated at 1300 °C. Changing temperature has little effect on the speciation when referenced to NNO. In Fig. 13b we show the speciation calculated using the values of $E_{V2/5}^*$, $E_{V3/5}^*$ and $E_{V4/5}^*$ in Table 2. The difference to Borisov (2013) is striking, particularly in the low abundance of V^4+^, suggesting that V redox speciation changes significantly from sodium disilicate melt to Fe-bearing natural melts.

**Fig. 14:**
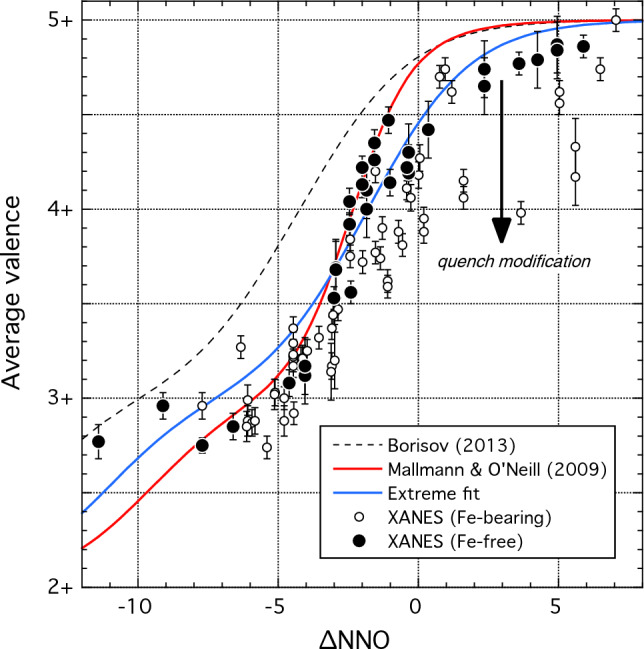
Vanadium average valence (V*) plotted versus ∆NNO. Open and filled circles denote XANES analyses of quenched Fe-bearing and Fe-free glasses respectively (Sutton et al. 2005; Righter et al. 2006, 2011). Dashed black line denotes calculated V* using redox potentials of Borisov (2013); red solid line uses redox potentials of Mallmann and O’Neill (2009); blue solid line uses extreme-fit redox potentials (Fig. 13c). Arrow denotes direction and magnitude of quench modification effects in Fe-bearing glasses as estimated by Borisov (2013)

Additional speciation information is available from XANES measurement of quenched glasses (Sutton et al. 2005). These data provide the pre-edge peak intensity of the vanadium K-edge XANES spectrum to obtain information on the average valence (V*), given as:

$$V^* = 5 \cdot X_{V^{5 + } } + 4 \cdot X_{V^{4 + } } + 3 \cdot X_{V^{3 + } } + 2 \cdot X_{V^{2 + } }$$

where $X_{V^{5 + } }$, $X_{V^{4 + } }$ etc. are the fractions of each species in the glass normalised to total V content. V* for 98 quenched glasses from a wide range of Fe-free and Fe-bearing glasses (Sutton et al. 2005; Righter et al. 2006, 2011) are plotted in Fig. 14. Although there is an expected increase in V* with increasing *f*O_2_ that data are very scattered. Generally, data for Fe-free glasses lie at higher V* at a given ∆NNO than do Fe-bearing glasses with the offset (and scatter) increasing with increasing *f*O_2_. The XANES data on V speciation are compared to the V* values obtained from Borisov (2013) and calculated from partitioning (values of $E_{V2/5}^*$, $E_{V3/5}^*$ and $E_{V4/5}^*$ from Table 2) in Fig. 14. The Borisov (2013) values do not match the XANES data, whereas the partitioning-based data give good agreement with the Fe-free glasses, but always lie to higher V* than the Fe-bearing glasses.

To test to what extent the mismatch between the partitioning based V* estimates and those from XANES data is simply an artefact of the fitting to the partitioning data, we have explored the limits of the range of $E_{V2/5}^*$, $E_{V3/5}^*$ and $E_{V4/5}^*$ that could explain the partitioning data. This is a useful exercise because the fit parameters obtained by Mallmann and O’Neill (2009) have, of necessity, high intercorrelation. The most extreme values of $E_{V2/5}^*$, $E_{V3/5}^*$ and $E_{V4/5}^*$ are defined by the point at which partition coefficients for individual valences (especially V^2+^ and V^4+^) become negative, a physical implausibility. The most extreme fit values of $E_{V2/5}^*$, $E_{V3/5}^*$ and $E_{V4/5}^*$ are –3.7, –0.9 and –0.1 respectively. Using these values moves V* to systematically lower values in line with the Fe-free XANES data (Fig. 14). The resulting V redox speciation is shown in Fig. 13c. Although the revised V* in Fig. 14 does move to lower values, it remain inconsistent with the low V* of many Fe-bearing glasses. Significantly, the extreme fit values of $E_{V2/5}^*$, $E_{V3/5}^*$ and $E_{V4/5}^*$ give a much closer match to the Fe-free, sodium disilicate redox speciation of Borisov (2013), albeit the peaks for each species are displaced to higher *f*O_2_ by approximately 2–3 log units in ∆NNO (Fig. 13c). These findings support the proposal of Borisov (2013) that homogenous reactions occur in Fe-bearing melts during quench, modifying the redox speciation such that the values measured in quenched Fe-bearing glasses using XANES do not necessarily correspond to those present in melt at high temperature. Borisov suggests that a reduction in V* by 0.3–0.4 is possible, which is entirely consistent with Fig. 14. In this context it is worth noting that some of the komatiitic XANES glasses studied by Sutton et al. (2005) are taken from olivine-melt partitioning experiments of Canil (1997) plotted in Fig. 10. It is not possible to reconcile the vanadium partitioning behaviour of olivines in Fig. 10 with these low V* values obtained via XANES indicating a change of V speciation during quench. Quench modification of vanadium is not an issue for crystal-melt partitioning studies because the speciation is unlikely to modify partitioning behaviour on quench timescales. Thus, for Fe-bearing systems mineral-melt partitioning of redox-sensitive elements such as vanadium may be a more reliable indicator of high-temperature melt speciation than XANES analyses of quenched glasses containing iron or other polyvalent elements, such as sulfur.

## Conclusions

In basaltic magma, *f*O_2_ exerts a strong control on the concentration of elements with multiple valences (e.g. Fe, Cr, V, Eu) and affects the stability (saturation temperature, modal abundance) of their host phases (i.e. spinel, pyroxene, olivine, plagioclase). Our experiments show that ülvospinel, pigeonite and clinopyroxene (and plagioclase) stability are increased under oxidized condition, at the expense of olivine, Cr-spinel and melt. Under oxidizing conditions, melt Fe^3+^/Fe_tot_ is high, enhancing Fe^3+^ + Al^IV^ exchange for Si + Mg in clinopyroxene. Along with increased spinel stability and modal abundance, this leads to a melt evolution trend from a tholeiitic to a more polymerized, quartz-normative calc-alkaline trend. Higher clinopyroxene Al^IV^ content allows more REE^3+^ to be incorporated onto the large M2 divalent site and increases the net fraction of suitably charged M2 site according to electrostatic energy considerations, as evidenced by sector zoned clinopyroxenes. Overall, however, the role played by *f*O_2_ on clinopyroxene-melt *D*_*REE*_ is indirect and negligible (except for Eu), in comparison to other factors such as temperature and chemical differentiation that also affect Al^IV^ content. Thus, there is no need to include explicitly *f*O_2_ in predictive thermodynamic models for *D*_REE_ based on clinopyroxene chemistry.

Concentrations and partitioning of multivalent elements in glass and minerals provide information about the *f*O_2_ condition at the time of crystallization. Optimal oxybarometers should be temperature-, pressure- and composition-independent. Based on our new experimental results and literature data, we explore the potential use of trace element oxybarometers based on mineral-melt partitioning. We show that olivine-melt *D*_V_, clinopyroxene-melt *D*_V_/*D*_Sc_ and plagioclase-melt *D*_Eu_/*D*_Sr_ all have potential as oxybarometers. The crystal chemical sensitivity of heterovalent cation incorporation into in clinopyroxene and the melt compositional sensitivity of the Eu^2+^-Eu^3+^ redox potential compromise this potential for clinopyroxene-melt and plagioclase-melt oxybarometers. However, olivine-melt *D*_V_ affords considerable precision and accuracy as an oxybarometer that is independent of temperature, and crystal and melt composition, as noted in several previous experimental studies,.

Variation of *D*_V_ and *D*_V_/*D*_Sc_ with *f*O_2_, normalised to NNO, for olivine and clinopyroxene contains information on the redox speciation of V in coexisting melt. By comparing the redox speciation constraints from partitioning to data from Fe-free synthetic systems and XANES spectroscopy of quenched glasses, we show that homogenous equilibria involving Fe and V species appear to modify the speciation of V during quenching, leading to a net overall reduction in the average vanadium valence (V*). Instead, mineral-melt partitioning of polyvalent species can provide a useful probe of redox speciation in Fe-bearing systems that is unaffected by quench effects. The same is also true for sulfur-bearing silicate melts where quench-related redox reactions may also be significant. Further experiments, over a wide range of *f*O_2_ are required if trace element partitioning is to be used to explore the compositional systematics of redox potentials in silicate melts.

### Supplementary Information

Below is the link to the electronic supplementary material.

Supplementary file1 (XLSX 36 KB)

Supplementary file2 (XLSX 16 KB)

Supplementary file3 (XLSX 367 KB)

Supplementary file4 (PDF 62 KB)

Supplementary file5 (EPS 1241 KB)

Supplementary file6 (XLSX 79 KB)

## Data Availability

The authors confirm that the data supporting the findings in this study are available within the article and the supplementary materials (ESM_1–6).

## Footnotes

1 Mg# = molar Mg/[Mg + Fe^2+^ + Fe^3+^].

2 Cr# = molar Cr/[Cr + Al + Fe^3+^].

3 Eu/Eu* = Eu_N_/[(Sm_N_ + Gd_N_)/2].
