# Supplementary figures and images for: Trace element partitioning in basaltic systems as a function of oxygen fugacity
Source: Contrib Mineral Petrol. 2023 Nov 27;178(12):95. doi: 10.1007/s00410-023-02069-x (PMC11008077; doi:10.1007/s00410-023-02069-x)

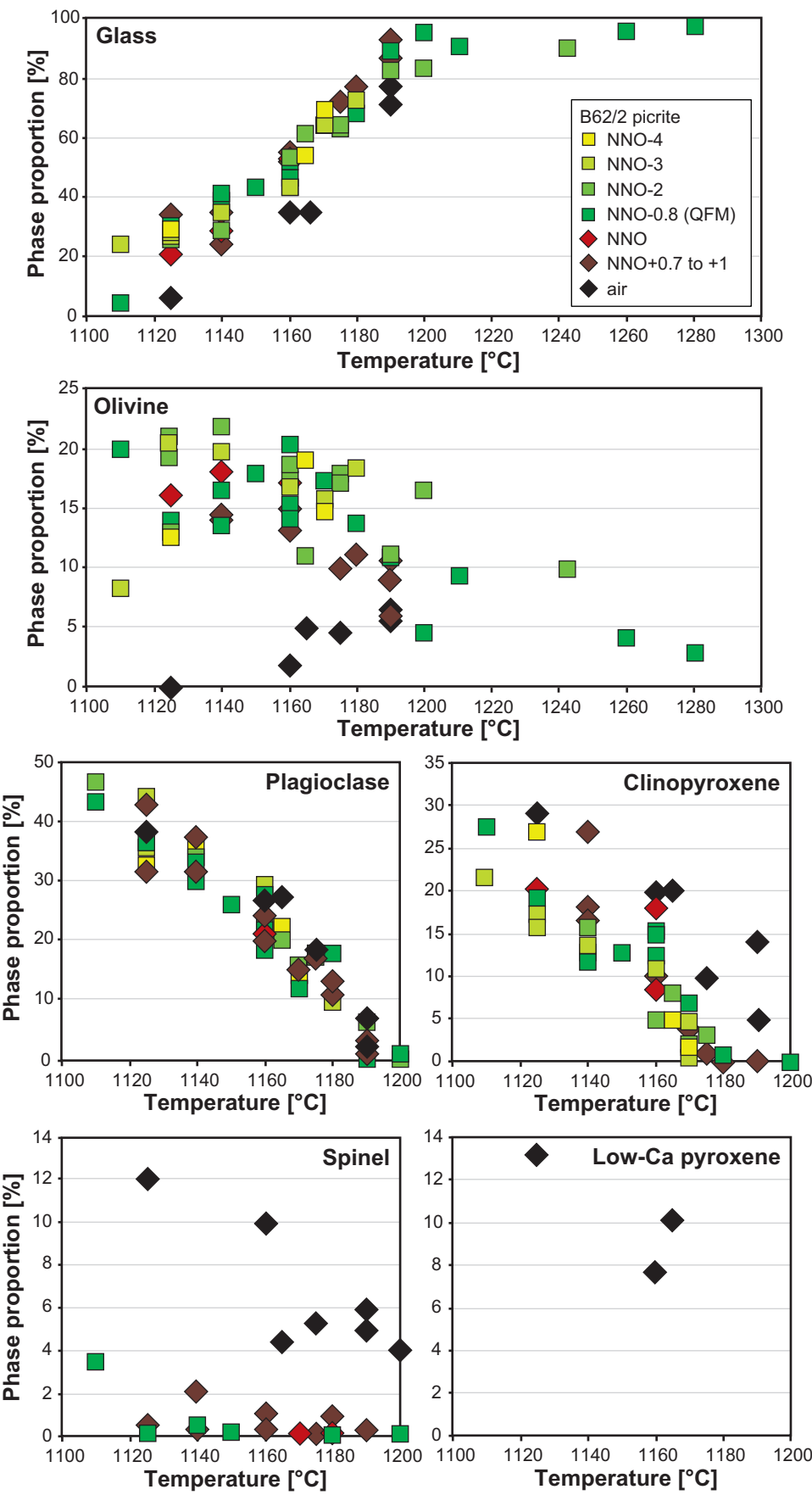

Supplement: Supplementary file 4 — Supplementary file4 (PDF 62 KB) [file 410_2023_2069_MOESM4_ESM.pdf]
